# ZEB1 Regulates Multiple Oncogenic Components Involved in Uveal Melanoma Progression

**DOI:** 10.1038/s41598-017-00079-x

**Published:** 2017-03-03

**Authors:** Yao Chen, Xiaoqin Lu, Diego E. Montoya-Durango, Yu-Hua Liu, Kevin C. Dean, Douglas S. Darling, Henry J. Kaplan, Douglas C. Dean, Ling Gao, Yongqing Liu

**Affiliations:** 1 The Second Xiangya Hospital, Central South University, Changsha, Hunan Province 410011 China; 2 Department of Ophthalmology and Visual Sciences, University of Louisville, Louisville, Kentucky USA; 3 Periodontics, Endodontics, and Dental Hygiene, University of Louisville, Louisville, Kentucky USA; 4 James Graham Brown Cancer Center, University of Louisville, Louisville, Kentucky USA

## Abstract

Human uveal melanoma (UM) is a major ocular malignant tumor with high risk of metastasis and requires multiple oncogenic factors for progression. ZEB1 is a zinc finger E-box binding transcription factor known for participating epithelial-mesenchymal transition (EMT), a critical cellular event for metastasis of malignant tumors of epithelium origin. ZEB1 is also expressed in UM and high expression of ZEB1 correlates with UM advancement, but has little effect on cell morphology. We show that spindle UM cells can become epithelioid but not vice versa; and ZEB1 exerts its tumorigenic effects by promoting cell dedifferentiation, proliferation, invasiveness, and dissemination. We provide evidence that ZEB1 binds not only to repress critical genes involving in pigment synthesis, mitosis, adherent junctions, but also to transactivate genes involving in matrix degradation and cellular locomotion to propel UM progression towards metastasis. We conclude that ZEB1 is a major oncogenic factor required for UM progression and could be a potential therapeutic target for treating UM in the clinic.

## Introduction

ZEB1 is an important transcription factor (TF) in development as deficiency in ZEB1 causes numerous birth defects including cleft palate, T cell scarcity, posterior cornea dystrophy, and even fetal death^1–3^. However, overexpression of ZEB1 has been discovered in many malignant tumors and positively correlated with their malignancy particularly in epithelium-derived carcinomas such as breast and lung cancers^4, 5^. As ZEB1 is an epithelial-mesenchymal transition (EMT) TF that directs epithelial cells to a more proliferative and mobile mesenchymal phenotype in development, its effects on tumorigenesis are thought to relate to this EMT^6–8^. ZEB1 can bind either to transactivate or to repress target genes through association with distinct partners such as co-activator P300 and co-repressor CtBP, respectively^9^. In EMT, ZEB1 represses the epithelial marker E-cadherin (*CDH1*) to weaken cell-cell adhesion, but enhances the mesenchymal marker N-cadherin (*CDH2*) to facilitate tumor progression by promoting cell migration and invasiveness^6, 8–12^.

EMT in other solid tumors besides carcinomas has not been widely reported perhaps because they are non-epithelium origin such as uveal melanoma (UM), derived from normal uveal melanocyte (NUM)^13, 14^. UM is a malignant intraocular tumor with over 50% of patients at risk of metastases, mostly are fatal^15^. Unlike carcinoma where EMT-TFs like SNAI, ZEB, and TWIST families are critical in tumorigenesis by switching the tumor towards mesenchymal phenotype, the spindle (mesenchymal-like) phenotype of UM is known for decades as a clinicopathologic factor indicative of decreased malignancy as compared to the aggressive epithelioid (epithelial-like) phenotype^16–18^. But, the questions of how and why these two phenotypes are induced over the course of UM progression have never been addressed experimentally. The reverse switch from EMT to MET (mesenchymal-epithelial transition) promotes cell dedifferentiation in cutaneous melanomagenesis^19^, and is correlated to the repression of *ID2* gene in the aggressive UM class^14^. Whether or not the EMT-TFs are involved in UM MET switch is currently not clear. In cutaneous melanomas, a molecular switch from ZEB2^high^/SNAI2^high^ to ZEB1^high^/TWIST1^high^ expression pattern is related to tumor initiation and progression^19^. In fact, both *TWIST1* and *ZEB1* are also reported to express higher in the aggressive UM class^14, 20^.

It appears that EMT-TFs are important for UM tumorigenesis and progression but not necessarily through EMT morphology switch. We hypothesize that these EMT-TFs and other factors regulate EMT morphology and tumor progression independently through distinct pathways and their combined action results in UM transformation and progression regardless of EMT morphology manifestation. Here we provide evidence that spindle UM cells can convert to epithelioid UM cells both *in vivo* and *in vitro* and that higher levels of ZEB1 propel UM progression by promoting cell dedifferentiation, proliferation, local migration and invasion, and distant dissemination though has little effect on EMT morphology. We conclude that ZEB1 is an oncogenic factor required for UM growth and metastasis.

## Results

### Epithelioid C918 cells are more aggressive than spindle OCM1 cells

**Figure 1:**
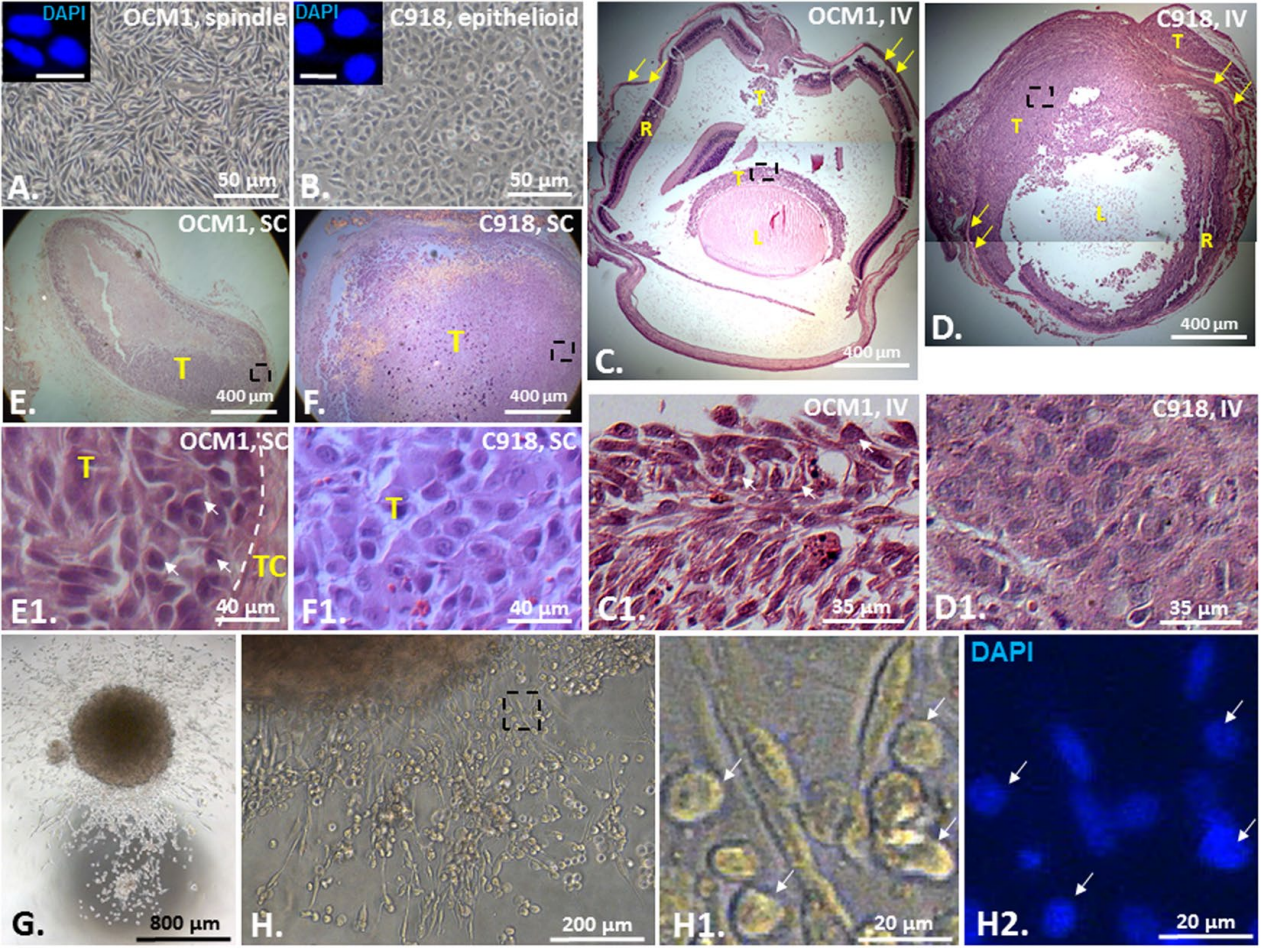
Epithelioid C918-derived tumors are more malignant than spindle OCM1-derived tumors. Confluent monolayer cultures of (**A**) the spindle UM cell line OCM1 and (**B**) the epithelioid UM cell line C918. Scale bar = 10 μm. Representative H&E stained images of both (**C**) OCM1- and (**D**) C918-derived ocular tumors generated in 13 days after IV injection, and both (**E**) OCM1- and (**F**) C918-derived cutaneous tumors generated in 13 days after SC injection. (**G**) A single OCM1 cell-formed sphere touched down and cells migrated out of the sphere. (**H**) OCM1 sphere-derived polymorphic cell types (inserts) including those with rounded epithelioid/amoeboid shape (arrows). IV = Intravitreal injection; SC = subcutaneous injection; L = Lens; R = Retina; T = Tumor; TC = Tumor Capsule. Yellow arrows indicate the sclera. White arrows indicate epithelioid cells in the OCM1-derived tumors and from an OCM1 sphere. Black dashed rectangles indicate the bottom-side inserts. Mean (M) ± standard deviation (SD).

In general, epithelioid UM is considered to be more aggressive than spindle UM^18, 21, 22^. To validate the claim we selected two widely-used and well-validated UM cell lines—spindle OCM1 (Fig. 1A) and epithelioid C918 (Fig. 1B)^23^ and implanted their suspended cells into the vitreous (IV) and subcutaneously (SC) into the rear flanks of the athymic nude mice to evaluate their malignant properties *in vivo* before investigating the underlying mechanism. As expected, C918 cells generated larger tumors than OCM1 cells in the grafted eyes and subcutaneous foci (Fig. 1C‒F). Within 13 days after grafting, C918-derived tumors (T) completely disrupted the eye structure though the residual remnants of the lens (L), the retina (R), and the sclera (arrows) were still visible (Fig. 1D, insert 1D1). By contrast, OCM1-derived tumors were still very small in the vitreous and the eye structure remained intact (Fig. 1C, insert 1C1). These observations suggest that the rapid growing C918-derived tumors might reduce nutrient supply to the normal eye tissues and aggressively invade into the nearby normal tissues, resulting in degeneration or resolving of the eye. However, the subcutaneously grafted tumors were all capsulized and no local invasion was found though C918-grafted tumors manifested larger than OCM1-grafted ones (Fig. 1F,G, inserts 1F1, 1G1), suggesting that the growth of the UM cells under the skin would be more restricted than in the eye. The liver metastases were revealed in the C918-grafted mice within 25 days as we will report later. Taken together, the epithelioid C918 cells are more aggressive *in vivo* than the spindle OCM1 cells.

### The spindle OCM1 cells can generate epithelioid/amoeboid cells after tumor formation *in vivo* or sphere formation *in vitro*

UM cells are transformed from the fibroblast-looking normal uveal melanocytes (NUMs). No research has been done in clarifying how NUMs are transformed into UM cells morphologically. In the clinic, UMs are classified histopathologically as spindle with elongated nuclei, epithelioid with more polygonal cytoplasm and pleomorphic nuclei, and mixed phenotypes^18, 24, 25^. The relationship between spindle and epithelioid phenotypes is not known though it is logical to speculate that a NUM would be firstly transformed into a spindle UM precursor and then developed to epithelioid phenotype as the UM progresses from non-aggressive to aggressive entity. To test this hypothesis we first checked cell morphology of the grafted tumors derived from OCM1 and C918. The H&E staining of the grafted tumor sections clearly showed that the OCM1-derived tumors consisted of both spindle and epithelioid cells (Fig. 1C1,E1) while C918-derived tumors were composed of only epithelioid cells (Fig. 1D1,F1) though these two UM cell lines were morphologically stable in monolayer culture (Fig. 1A,B and inserts), suggesting that spindle cells could become epithelioid in grafted tumors but not vice versa. Similarly, we also found that a single OCM1 cell-derived sphere in suspension culture (Fig. 1G) gave rise to large number of fast moving and rounded epithelioid or amoeboid (the term amoeboid migration is defined predominantly by the cellular morphology)^26^ cells as recently discovered in cutaneous melanoma^27, 28^ (Fig. 1H), validating that spindle cells are morphologically flexible *in vitro* as well.

### High expression of *ZEB1* is linked to UM malignancy

**Figure 2:**
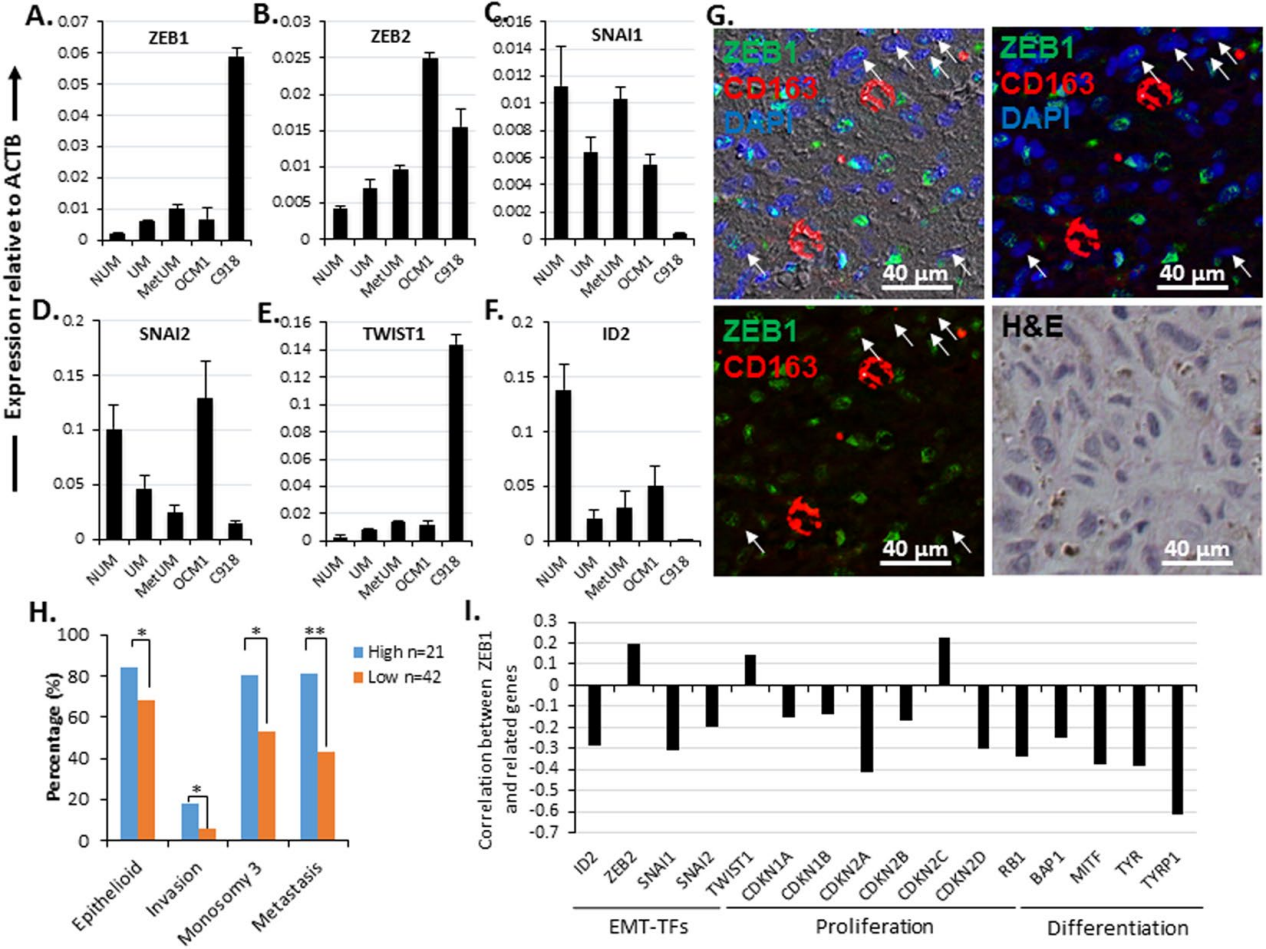
Expression of EMT-TFs in human NUM, UM, MetUM, OCM1 and C918 cells. Transcriptional levels of EMT-TFs were examined in NUM, UM, MetUM by microarrays^20, 21^, and in OCM1, and C918 by qPCR. The expression levels of individual genes were normalized to that of the house-keeping gene β-actin (*ACTB*). (**A**) *ZEB1*; (**B**) *ZEB2*; (**C**) *SNAI1*; (**D**) *SNAI2*; (**E**) *TWIST1*; (**F**) *ID2*. (**G**) A representative patient UM of mixed phenotype showing ZEB1-stained nuclei are not elongated spindle shape and a few CD163 positive but ZEB1 negative thereby potential macrophages are scattered in the tumor. Arrows indicate elongated spindle nuclei which are ZEB1 negative and an H&E-stained section of the same area is included to show the mixed phenotype. (**H**) Statistical analyses of a large cohort of total 63 primary UMs (GES22138, Supplemental Table 4) on a number of selected tumor malignant properties in relation to their *ZEB1* expression levels. After sorted from high to low expression of *ZEB1*, based on preliminary assessment we assign the top one third of the UMs as *ZEB1* ^high^ (n = 21) and the bottom two third of the UMs as *ZEB1* ^low^ (n = 42). (**I**) Correlation coefficients are calculated based on the microarray data set (GES22138), showing that expression of critical genes involved in EMT, cell proliferation and differentiation is correlated to that of *ZEB1*. Mean (M) ± standard deviation (SD). ‘*’ Indicates Student’s t-test *p* ≤ 0.05, ‘**’ Indicates Student’s t-test *p* ≤ 0.01.

Epithelium-originated cancer cells tend to be of higher malignancy when having transited to be mesenchymal morphology like in breast and lung cancers^4, 5^. This EMT is primarily modulated by a group of E-box binding TFs including SNAI, TWIST, and ZEB families and can serve as a poor prognosis marker^9, 13^. These EMT-TFs also play an important role in progression of non-epithelium-derived tumors^29, 30^ including melanomas^19, 20^. A molecular switch from SNAI2^high^/TWIST2^high^ to ZEB1^high^/TWIST1^high^ is discovered to determine transformation of skin melanocytes to cutaneous melanoma cells^19^. To validate if this switch also happens in UM, we checked the expression of E-box binding TF families, plus the known E-cadherin repressor *ID2* ^14^ in NUM, UM and metastatic UM (MetUM) using two microarray data sets (GSE62075 for NUM^31^ and GSE39717 for UM and MetUM^32^, see detail in Methods). Indeed, *ZEB* family and *TWIST1* were expressed high in UM and even higher in MetUM compared to NUM (Fig. 2A,B,E), whereas *SNAI2* and *ID2* expressed lower in UM and MetUM (Fig. 2D,F). There were no significant change in expression of *SNAI1* (Fig. 2C) and no detection of *TWIST2*. There was a switch from *ZEB1* ^low^/*TWIST1* ^low^/*SNAI* ^high^/*ID2* ^high^ in the non-aggressive spindle OCM1 cells to *ZEB1* ^high^/*TWIST1* ^high^/*SNAI* ^low^/*ID2* ^low^ in the aggressive epithelioid C918 cells (Fig. 2A,C‒F). Immunostaining with ZEB1 antibody on a patient UM of the mixed cell type confirmed that ZEB1 was only present in the epithelioid cells (Figs 2G and 3E) that was negative for CD163, a marker for tumor-associated macrophages^33, 34^ (Fig. 2G). Apparently, *ZEB1* was expressed high in UM (Fig. 2A)^20^; and its high expression appeared to be associated with malignancy but not with mesenchymal (spindle) phenotype in UM (Fig. 2A,G). To validate whether *ZEB1* expression levels of UM truly reflect their histopathological classifications and malignant properties, we downloaded a full set of microarray data of total 63 primary UMs (GSE22138) together with tumor characteristics information including tumor cell type, scleral invasion, monosomy 3, and metastasis (see detail in Methods)^24^. We sorted all tumors from high to low based on their *ZEB1* expression levels, and ‘equally’ divided them into either two groups (high and low) or three groups (high, medium, low). We found that the positive correlation of *ZEB1* expression with the patient metastasis was less significant for the two-group division while it was very significant for the three-group division though no significance was detected between the medium and low groups (data not shown). Based on this assessment we pooled the medium and low groups into a new low group: the top one third of *ZEB1* ^high^ UMs and the other bottom two third of *ZEB1* ^low^ UMs (Supplemental Table 4). As a result, high expression of *ZEB1* is indeed positively correlated to epithelioid phenotype and other malignant characteristics of UMs (Fig. 2H). Meanwhile, *ZEB1* expression levels also correlate positively to that of *ZEB2* and *TWIST1*, but negatively to that of *ID2* and *SNAI* family (Fig. 2I), suggesting a potential regulation of ZEB1 on the molecular switch from *ZEB1* ^low^/*TWIST1* ^low^/*SNAI* ^high^/*ID2* ^high^ to *ZEB1* ^high^/*TWIST1* ^high^/*SNAI* ^low^/*ID2* ^low^ in UMs.

**Figure 4:**
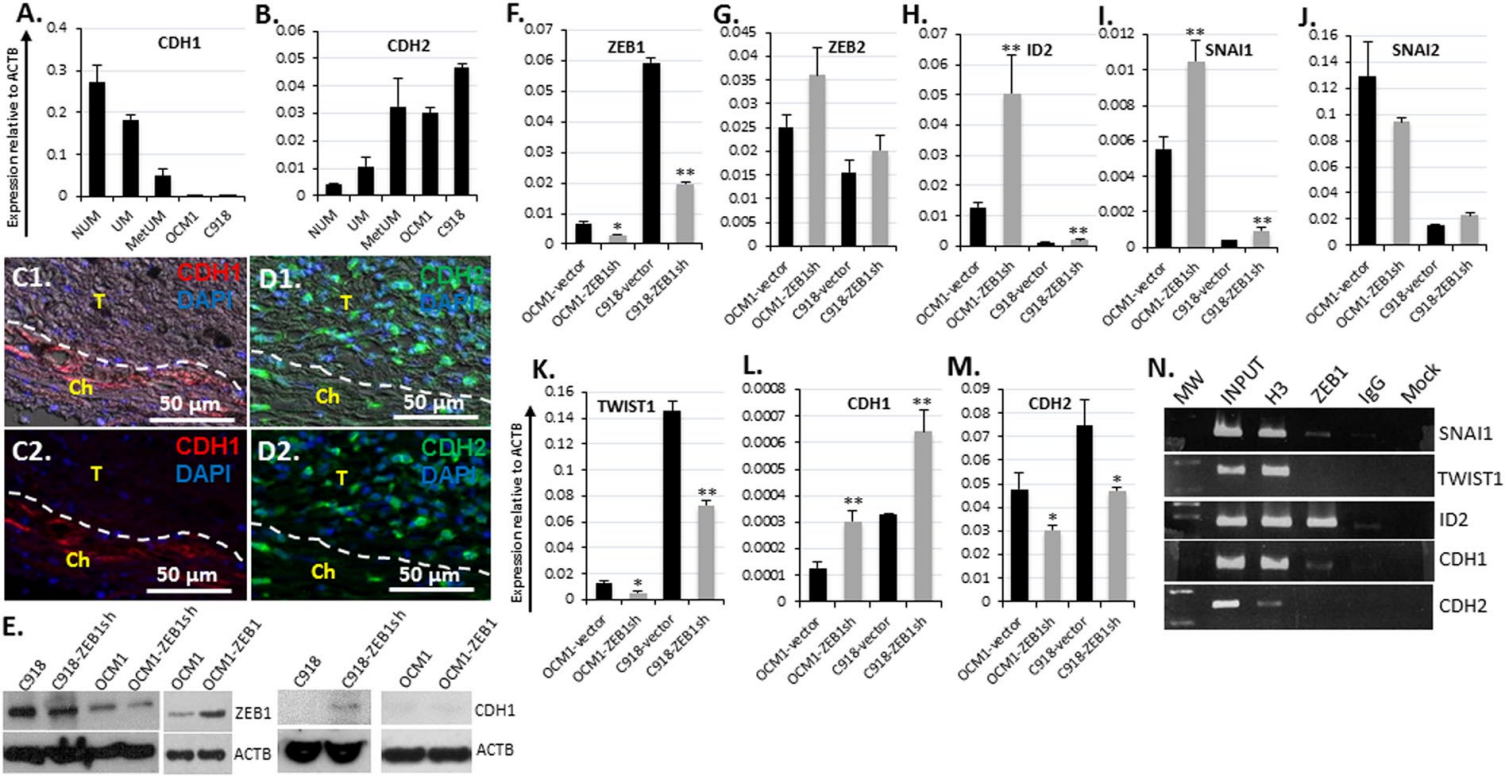
ZEB1 promotes UM cell dedifferentiation by repressing the pigment synthesis pathway. A significant underexpression of genes involving in melanocyte differentiation such as (**A**) *BAP1*, (**B**) *MITF*, (**C**) *TYR*, and (**D**) *TYRP1* was detected in UM and MetUM as compared to NUM. Higher expression of these differentiation genes was also detected in the spindle non-aggressive OCM1 as compared to the epithelioid aggressive C918. (**E**) ZEB1 was only detected in the epithelioid cells which manifested non- or less pigment in a representative patient UM section. An H&E stained section of the same area is included to show the mixed phenotype. Knockdown of ZEB1 significantly amplified transcription of all these differentiation genes in both (**F**) OCM1 and (**G**) C918. (**H**) ChIP assays showed that ZEB1 bound to *MITF-M* (melanocyte-specific) and *BAP1* promoters, likely to repress their expression. Mean (M) ± standard deviation (SD). ‘*’ Indicates Student’s t-test *p* ≤ 0.05, ‘**’ Indicates Student’s t-test *p* ≤ 0.01.

**Figure 3:**
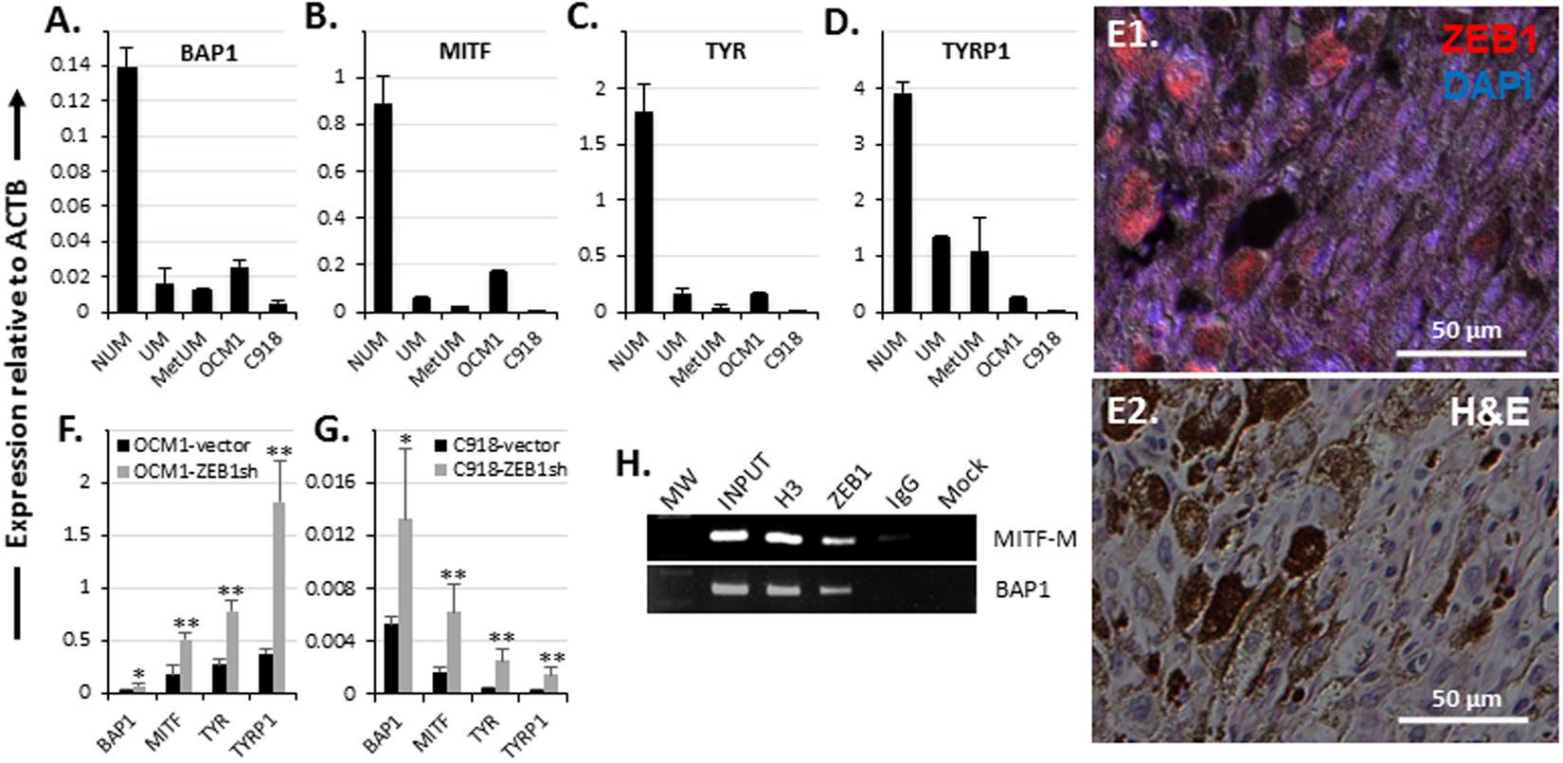
ZEB1 coordinates other EMT-TFs in transcriptional regulation of EMT molecules. Transcriptional expression levels of (**A**) the epithelial marker E-cadherin (*CDH1*), (**B**) the mesenchymal marker N-cadherin (*CDH2*) detected by qPCR in NUM, UM, MetUM, OCM1 and C918 were compared. The presence of (**C**) CDH1 in the choroidal tissue but not in the tumor, and **(D)** more CDH2 in the tumor than in the choroid was detected by immunohistochemistry. (**E**) ZEB1 knockdown in both C918 and OCM1 and ZEB1 overexpression in OCM1 were validated by Western blots (WB). Knockdown of ZEB1 resulted in CDH1 accumulation in C918. Real-time qPCR analyses of the effects of (**F**) *ZEB1* knockdown on the expression of the other EMT-TF genes including (**G**) *ZEB2*, (**H**) *ID2*, (**I**) *SNAI1*, (**J**) *SNAI2*, (**K**) *TWIST1*, and EMT marker genes including (**L**) *CDH1*, and (**M**) *CDH2*. (**N**) Chromatin immune-precipitated (ChIP) by ZEB1 antiserum (ZEB1) was utilized to detect the binding of ZEB1 to the EMT-TF genes *SNAI1*, *TWIST1* and *ID2*, and to the EMT marker genes *CDH1* and *CDH2*. Molecular weight (MW) marks were loaded on the far left lane for validation of ChIP-PCR amplicon size, followed by 10x diluted chromatin sample before immuneprecipitation (INPUT). Pan histone 3 (H3) and pre-immune serum (IgG) were served as a positive and a negative control, respectively, whereas any remaining chromatin after passing through the binding beads without any serum was served as a background control (Mock). Mean (M) ± standard deviation (SD). ‘*’ Indicates Student’s t-test *p* ≤ 0.05, ‘**’ Indicates Student’s t-test *p* ≤ 0.01.

Since the figures were not cited in numerical order, they have been renumbered in the order of appearance. Please check and confirm.Figure 3 should switch back to figure 4 as originally indicated in the text, and change the cited number accordingly. Thanks!

### ZEB1 triggers EMT molecular switch but has little effect on cell morphology manifestation in Us

It is of note that UM is a non-epithelial cell-derived tumor and epithelioid UM cells are just epithelial-like, whether or not the expression of epithelial marker E-cadherin (CDH1), a tumor repressor for carcinomas, and mesenchymal cell markers like N-cadherin (CDH2), a tumor progression facilitator for carcinomas, also correlates with the malignancy of UM is not clear. To check the facts, we examined the transcription of these two major EMT marker genes in NUM, UM, and MetUM. As with carcinoma, the epithelial marker *CDH1* was downregulated in UM and down more in MetUM compared to the untransformed NUM (Fig. 3A), while the mesenchymal marker *CDH2* was upregulated in UM and up more in MetUM (Fig. 3B). In agreement with the transcriptional profile, immunostaining of patient tumor sections showed that CDH1 was only present in the choroidal tissue (Fig. 3 C) while CDH2 manifested an opposite pattern (Fig. 3D). These EMT markers appeared to have no influence on UM cell morphology as no CDH1 was detected in both the spindle OCM1 and the epithelioid C918 cells (Fig. 4A) and the epithelioid C918 expressed even higher level of mesenchymal markers *CDH2* than the spindle OCM1 though both of the UM cell lines expressed much higher levels of CDH2 as compared to NUM (Fig. 3B). These results suggest that UM cells are of mesenchymal characteristics regardless of their distinct morphology, and that downregulation of *CDH1* and upregulation of *CDH2* appear related to neoplastic transformation of NUM to UM and progression of UM towards MetUM (Fig. 3A,B).

### ZEB1 coordinates with other EMT-TFs to regulate EMT marker genes in UM

As demonstrated above, *ZEB1* was expressed higher in UM and MetUM as compared to NUM, and much higher in the aggressive epithelioid C918 than in the non-aggressive spindle OCM1 (Fig. 2A,E), suggesting that ZEB1 is a major EMT-TF that propel UM progression towards metastasis as it has been shown in carcinomas^11, 12^. To functionally analyze how ZEB1 regulates UM progression we overexpressed ZEB1 in ZEB1^low^ OCM1 cells (OCM1-ZEB1) and knocked it down in both ZEB1^high^ C918 (C918-ZEB1sh) and ZEB1^low^ OCM1 cells (OCM1-ZEB1sh) by transduction of a full-length human *ZEB1* gene and a short hairpin RNA (shRNA) against human *ZEB1* mRNA, respectively (see detail in Methods). Four lentiviral *ZEB1* shRNA and one scramble control constructs were purchased and viral particles were assembled in the laboratory^6^. Their infection efficiency was screened based the levels of EGFP expression and their *ZEB1* knockdown levels detected by qPCR in the infected C918 cells (data not shown), the viral construct with the *ZEB1*-specific interference nucleotide sequence 5′- AACAATACAAGAGGTTAAA -3′ was selected for further ZEB1 functional studies with OCM1 and C918 (Supplemental Table 1). Overexpression and downregulation of ZEB1 in OCM1 and C918 by the constructs were validated by both WB and qPCR (Fig. 3E,F). With knockdown of ZEB1, *ZEB2*, *ID2* and *SNAI1* were upregulated (Fig. 4G‒I) while *TWIST1* was significantly downregulated in both OCM1 and C918 (Fig. 3K). We did not observe any cell morphology change in OCM1-ZEB1 and in C918-ZEB1sh as compared to their parental cells though CDH1 showed upregulation in C918-ZEB1sh and OCM1-ZEB1sh (Fig. 3E,L). By contrast, knockdown of ZEB1 significantly reduced *CDH2* in the both cell lines (Fig. 4M). To check whether ZEB1 binds to the promoters of other EMT-TFs and EMT marker genes, we conducted chromatin immunoprecipitation (ChIP) assays with the ZEB1 antiserum^35^, and found that ZEB1 directly bound and thereby possibly repressed *SNAI1*, *ID2*, and *CDH1* genes (Fig. 3N). But, no binding of ZEB1 to the promoters of both *TWIST1* and *CDH2* was revealed (Fig. 3N), thereby how ZEB1 regulates these two genes remained elusive. Taken together, we concluded that ZEB1 plays a central role either directly or indirectly to regulate EMT marker genes in UM, but has little effect on directing UM cell to be mesenchymal morphology.

### ZEB1 represses transcription of differentiation genes in UM

An important sign of normal cell transformation into cancer cell is dedifferentiation. For example, compared to their parental epithelial cells, carcinoma cells express much less epithelial markers like CDH1^9^. Normal melanocytes are pigmented cells, genes involving in pigment synthesis like *MITF*, *TYR*, and *TYRP1* are typical differentiation makers for these cells^36, 37^, we therefore examined whether these pigment synthesis genes together with *BAP1*, another important differentiation gene whose loss-of-function mutation is frequently related to UM transformation^38^, would be downregulated in UM and MetUM as compared to NUM. All the tested genes were greatly decreased in expression in UM and in MetUM (Fig. 4A‒D), implying that UM transformation and progression to metastasis are paralleled with their cell dedifferentiation. Also as expected, these genes were expressed much higher in OCM1 than in C918 (Fig. 4A‒D), suggesting that dedifferentiation is positively related to UM cell malignant advancement. Immunostaining of a patient UM section with the antibody against ZEB1 demonstrated that ZEB1 was present mostly in non- or less pigmented epithelioid cells (Fig. 4E). In addition, *ZEB1* expression levels are negatively correlated to these differentiation genes in UMs (Fig. 2I), and knockdown of ZEB1 significantly reduced the expression of *BAP1*, *MITF*, *TYR*, and *TYRP1* (Fig. 4F‒G), suggesting that ZEB1 regulates their transcription. ChIP assays showed that ZEB1 directly bound to the promoters of both *BAP1* and *MITF-M* (a specific promoter for *MITF* to express in melanocyte)^36, 37^ (Fig. 4H) and thereby likely repressed their expression.

### ZEB1 promotes UM cell proliferation

**Figure 5:**
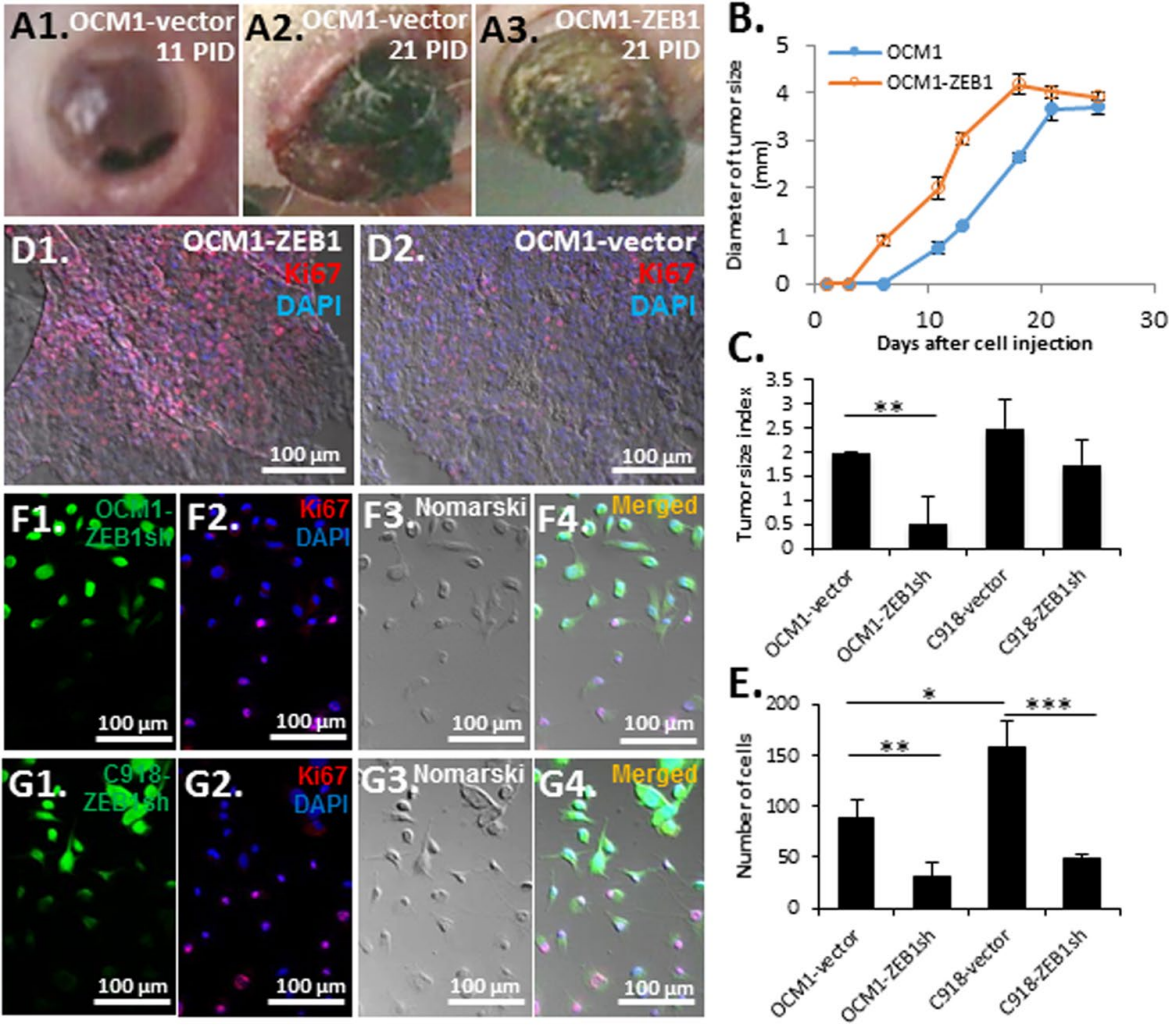
Effects of ZEB1 on tumor growth and cell proliferation. (**A**) Representative images showing tumor growth out of the eyes grafted with either OCM1-vector control or OCM1-ZEB1 cells at 11 and 21 post-inoculation day (PID). (**B**) OCM1-ZEB1 cell-derived tumors appeared 5 days earlier than OCM1 cell-derived tumors, but their growth rates and final tumor sizes were similar. (**C**) Equal number of cells (2 × 10^5^) of both OCM1- and C918-vector controls and ZEB1sh knockdown cells were intravitreously injected into the nude mice and their 25-day grafted tumors were examined for tumor size evaluation. Based on the tumor H&E-stained sections, tumor size were subjectively classified into ‘No’, ‘Small’, ‘Medium’, and ‘Big’ categories and digitalized as 0, 1, 2, and 3, respectively. The pooled result of total 4 tumors was presented as a tumor size index for each injected cell line (Supplemental Table 5). (**D**) More mitotic cells were detected by Ki67 immunostaining in OCM1-ZEB1 cell-derived tumors than in OCM1-vector cell-derived tumors. (**E**) Equal number of cells (1 × 10^4^) were seeded in a 10-cm culture plate. Cells in 5 randomly selected squares (5 × 5 mm^2^) were counted. Both (**F**) OCM1-ZEB1sh and (**G**) C918-ZEB1sh cells were cultured on a chamber slide and immunostained with the mitotic marker Ki67 (red) before confluence. As knockdown levels of ZEB1 were consistent with the expression of EGFP (green), EGFP-dim thereby ZEB1^higher^ cells showed more Ki67 positive than those EGFP-bright thereby ZEB1^lower^ cells. Mean (M) ± standard deviation (SD). Student’s t-tests were performed. *indicates *p* ≤ 0.05; ** Indicates *p* ≤ 0.01; *** Indicates *p* ≤ 0.001.

As demonstrated early, ZEB1^high^ C918-grafted tumors grew much larger than ZEB1^low^ OCM1-grafted tumors (Fig. 1C‒F), we speculated that ZEB1 might promote UM growth. Indeed upon overexpression of ZEB1, OCM1-ZEB1-grafted tumors became visible 5 days earlier than OCM1-vector control (Fig. 5A,B), while knockdown of ZEB1 significantly reduced the growth of OCM1-ZEB1sh-grafted tumors though C918-ZEB1sh-grafted tumors only manifested a slight decrease in tumor size as compared to vector controls (Fig. 5C, Supplemental Table 5). Immunostaining with the antibody against the mitotic marker Ki67 on the grafted tumor sections showed that there were many more proliferating cells in the OCM1-ZEB1 tumor than in the OCM1-vector control (Fig. 5D). In line with the *in vivo* data, knockdown of ZEB1 also significantly reduced the growth rates of the UM cell lines in culture (Fig. 5E). For the cultured OCM1-ZEB1sh and C918-ZEB1sh cells, though all selected by puromycin right after the ZEB1sh viral infection (the viral vector contains both puromycin resistance and EGFP genes) there were still about 20% cells with dim EGFP expression due to less viral insertion numbers thereby less downregulation of ZEB1 (Fig. 5F1,G1). As a result, the Ki67 positive cells were mostly those OCM1-ZEB1sh or C918-ZEB1sh cells with less EGFP thereby more ZEB1 in culture (Fig. 5F,G), suggesting that ZEB1 promotes cell proliferation.

### ZEB1 represses expression of cyclin-dependent kinase inhibitors (CDKIs)

**Figure 6:**
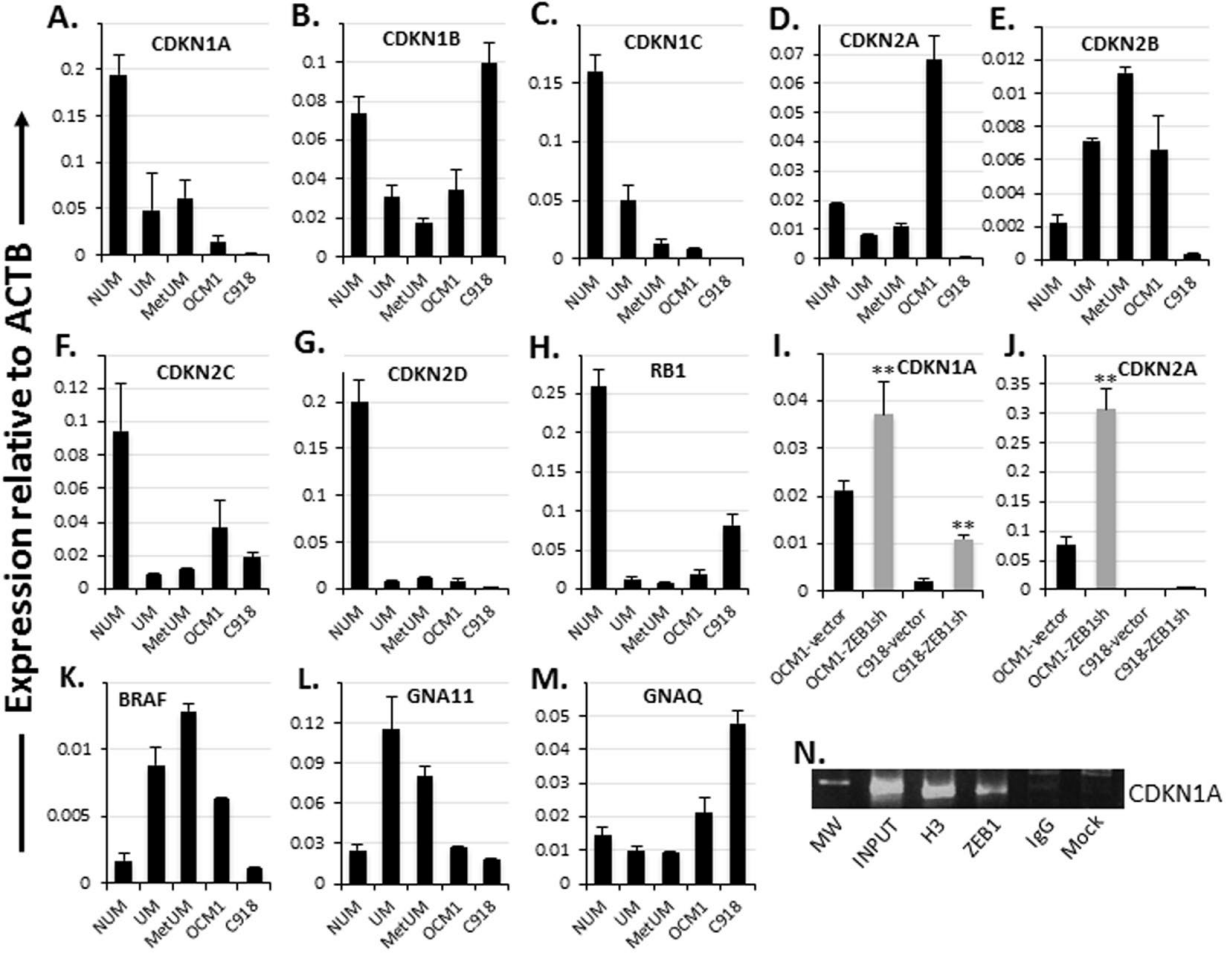
Expression and regulation of CDKIs and other cell cycling-related genes. Except for *CDKN2B*, transcriptional analyses showed that all other tested *CDKI* genes and the tumor suppressor *RB1* were underexpressed in UM and MetMU as compared to NUM; and all the *CDKI* genes, except for *CDKN1B*, were downregulated in C918 than in OCM1. (**A**) *CDKN1A*. (**B**) *CDKN1B*. (**C**) *CDKN1C*. (**D**) *CDKN2A*. (**E**) *CDKN2B*. (**F**) *CDKN2C*. (**G**) *CDKN2D*. (**H**) *RB1*. Knockdown of ZEB1 significantly magnified (**I**) *CDKN1A* and (**J**) *CDKN2A* expression in both OCM1 and C918. Some G-protein-related signaling elements such as (**K**) *BRAF* and (**L**) *GNA11* were transcriptionally upregulated in UM and MetUM, but not (**M**) *GNAQ*. (**N**) ChIP assays showed that ZEB1 bound the promoter of *CDKN1A*. Mean (M) ± standard deviation (SD). ‘**’ Indicates Student’s t-test *p* ≤ 0.01.

To examine how ZEB1 enhances UM cell division thereby tumor growth we checked the expression levels of cell cycling-related genes and found that all detected CDKIs, except for *CDKN2B* (*P15* ^*INK4B*^), including *CDKN1A* (*P21* ^*CIP1*^)*, CDKN1B* (*P27* ^*KIP1*^)*, CDKN2A* (*P16* ^*INK4A*^ */P14* ^*ARF*^)*, CDKN2C* (*P18* ^*INK4C*^), and *CDKN2D* (*P19* ^*INK4D*^) together with the nuclear phosphoprotein RB1 were underexpressed in UM and MetUM compared to NUM (Fig. 6A‒H). Consistently, negative correlations between *ZEB1* and these genes except for *CDKN2C* are found in UMs (Fig. 2I). Also, except for *CDKN1B*, the other CDKIs were expressed much higher in OCM1 than in C918 (Fig. 6A‒H), suggesting that the expression of CDKIs is possibly repressed by ZEB1. As speculated, knockdown of ZEB1 upregulated *CDKN1A* (*P21* ^*CIP1*^) and *CDKN2A* (*P16* ^*INK4A*^) (Fig. 6I and J), suggesting that both P16^INK4A^/RB1 and TP53/P21^CIP1^ tumor suppression pathways are deregulated with high expression of ZEB1 in UM cells leading to rapid cell proliferation with no risk of apoptotic cell death as downregulation of the above CDKIs would phosphorylate and inactivate RB1 so as to promote cell cycling^9^. It is known that Zeb1 binds and thereby represses Cdk inhibitor genes in mouse cells^8^. To check whether ZEB1 binds and represses *CDKN1A* and *CDKN2A* genes in human tumor cells, we conducted ChIP assays and found that ZEB1 was indeed bound to *CDKN1A* promoter (Fig. 6N), implying a direct regulatory role for ZEB1 to promote cell proliferation in UM cells. In addition, in melanomas of both uveal and cutaneous origins a G protein-related signaling pathway is frequently activated through a gain-of-function mutation of either large GTPase α subunit like GNA11 and GNAQ^39, 40^ or their homologous—small GTPase like NRAS and its downstream effector BRAF^41, 42^. Interestingly, we found that *BRAF* and *GNA11* were transcriptionally upregulated in UM and MetUM while *GNAQ* in both OCM1 and C918 and *BRAF* in OCM1 were also expressed higher as compared to NUM (Fig. 6K‒M), suggesting that although or GNA11/GNAQ or NRAS/BRAF mutations are often found in uveal and cutaneous melanomas, respectively^43, 44^, the overall elevated transcription levels of the above genes position UM cells in a high potential readiness for extracellular signals for cell proliferation regardless of their gain-of-function mutation status.

### ZEB1 facilitates UM cell invasiveness

**Figure 7:**
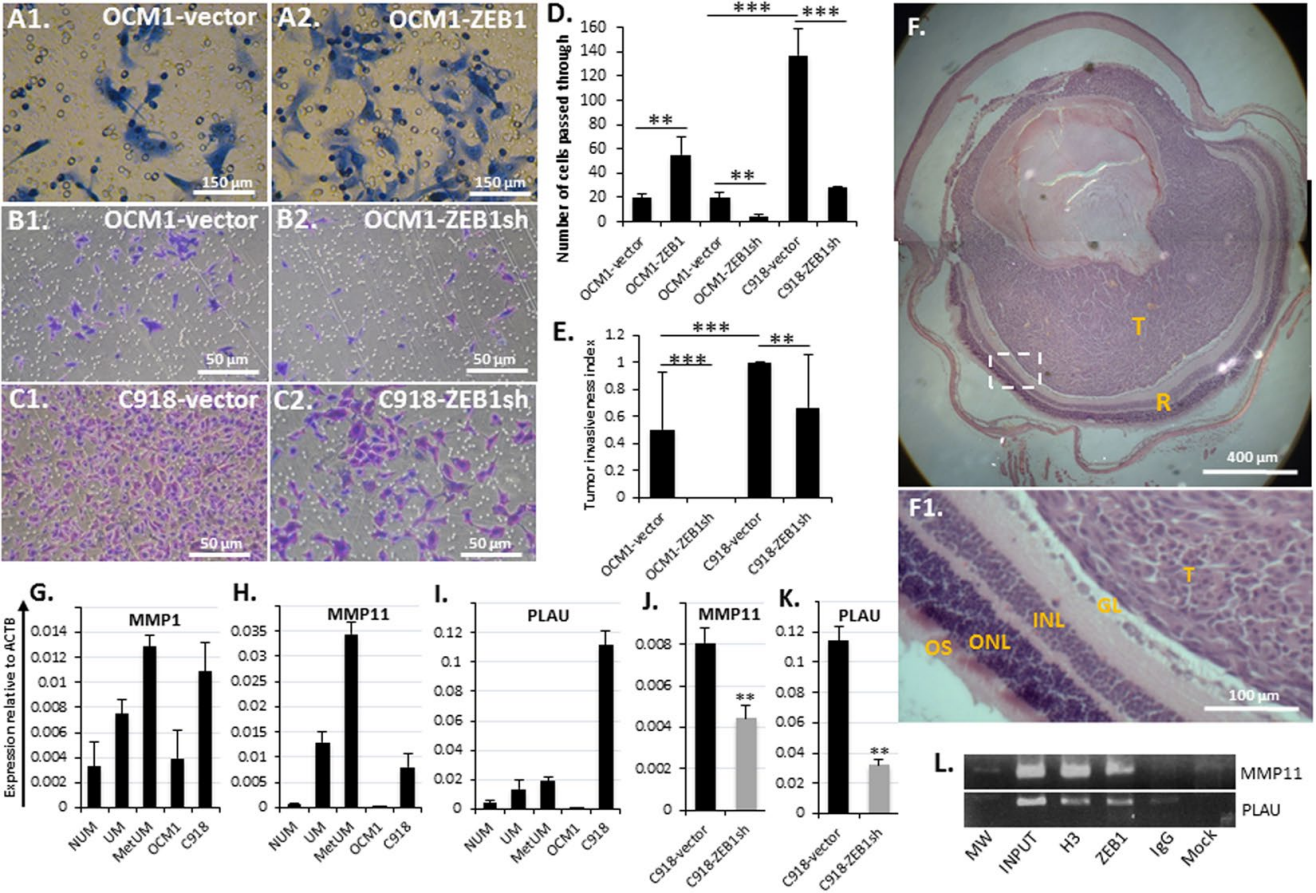
ZEB1 promotes UM cell invasiveness. Cells passed through the transwell membranes were formaldehyde fixed, crystal violet stained. (**A**) Representative images of the penetrated OCM1-vector control cells and ZEB1 overexpressed OCM1 cells (OCM1-ZEB1). (**B**) Representative images of the penetrated OCM1-vector control cells and ZEB1-knockdown OCM1 (OCM1-ZEB1sh). (**C**) Representative images of the penetrated C918-vector control cells and ZEB1-knockdown C918 (C918-ZEB1sh). (**D**) The number of the penetrated cells affected by ZEB1 overexpression or downregulation were counted in 4 randomly selected microscopic fields at day 2 after equal number cell seeding and a mitomycin C treatment. (**E**) Equal number of OCM1- and C918-vector controls and ZEB1sh knockdown cells (2 × 10^5^) were intravitreously injected into the nude mice and their 25-day grafted ocular tumors were examined for tumor invasiveness evaluation. (**F**) A representative H&E stained section of the OCM1-ZEB1sh cell-derived tumor in the grafted vitreous, showing that the tumor (T) was not invasive to the surrounding retina (R) including the ganglion layer (GL), inner nuclear layer (INL), outer nuclear layer (ONL), and the outer segment (OS). Transcriptional analyses of (**G**) matrix matelloproteinases *MMP1*, (**H**) *MMP11*, and (**I**) another matrix degradation proteinase *PLAU*. Knockdown of ZEB1 reduced both (**J**) *MMP11* and (**K**) *PLAU* expression in C918. (**L**) ChIP assays showed that ZEB1 bound to the *MMP11* and *PLAU* gene promoters. Mean (M) ± standard deviation (SD). Student’s t-tests were performed. ** Indicates *p* ≤ 0.01; *** Indicates *p* ≤ 0.001.

Tumor malignancy is attributed to the abilities of cancer cells not only to proliferate but also to invade locally and to disseminate in distance. As shown above, ZEB1^high^ C918-derived tumors destroyed the eye structure while ZEB1^low^ OCM1-derived tumors did not in 13 days after cell grafting (Fig. 1C,D), suggesting that ZEB1 might increase UM cell invasiveness as with that of carcinoma cells^11, 12^. To experimentally assess whether OCM1-ZEB1 cells exert a more invasive capacity than OCM1 cells *in vitro*, we adopted a transwell system to evaluate UM cell invasiveness in culture (see detail in Methods). As expected, more OCM1-ZEB1 cells penetrated the Matrigel-coated membrane than OCM1-vector control (Fig. 7A,D). Downregulation of ZEB1 significantly reduced UM cell ability to penetrate the membrane (Fig. 7B‒D), confirming that ZEB1 enhances tumor invasiveness. More importantly, knockdown of ZEB1 reduced or completely diminished the invasive capacity of C918 and OCM1 UM cells, respectively, in the grafted eyes within 25 days after the intravitreal injection (Fig. 7E,F, Supplemental Table 5). Furthermore, high expression of ZEB1 is shown to be significantly correlated to tumor scleral invasion and metastasis (Fig. 2H). In agreement with these observations, the expression of extracellular matrix digestion enzyme genes like *MMP1* and *MMP11* ^45^, and *PLAU* ^46^, was high in UM and even higher in MetUM as compared to NUM (Fig. 7G‒I). These proteinase genes were expressed much higher in ZEB1^high^ C918 than ZEB1^low^ OCM1 (Fig. 7G‒I). Downregulation of ZEB1 in C918 significantly reduced the expression of both *MMP11* and *PLAU* (Fig. 7J,K), suggesting that ZEB1 transactivate their transcription. ChIP assays showed that ZEB1 bound and likely upregulated both *MMP11* and *PLAU* genes (Fig. 7L).

### ZEB1 loosens UM cell adhesion and promotes UM metastases

**Figure 8:**
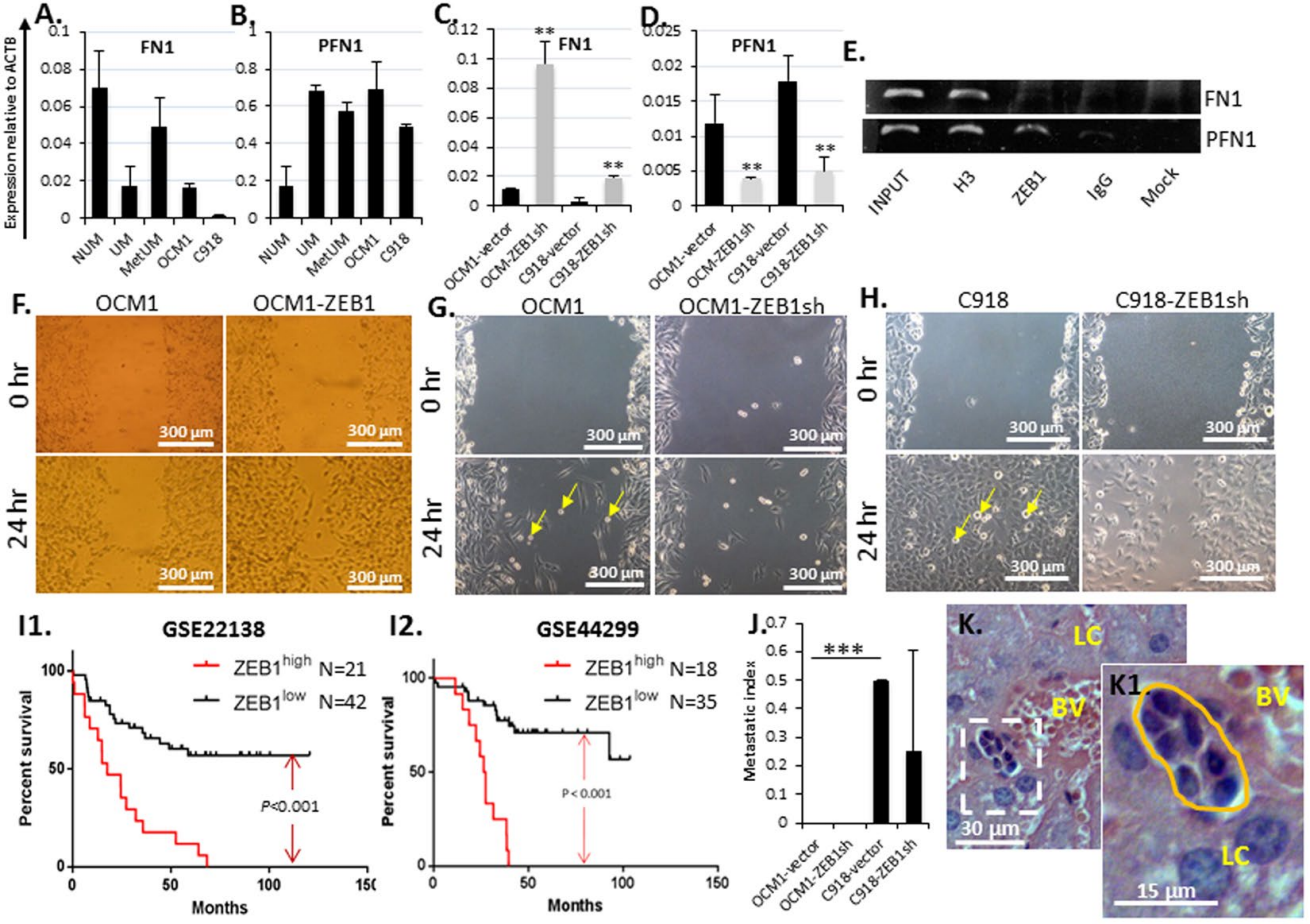
ZEB1 facilitates UM cell migration. Transcriptional analyses showed that (**A**) the major component of extracellular matrix (ECM) fibronectin (*FN1*) and (**B**) the locomotion protein profilin (*PFN1*) were under- or over-expressed in UM, MetUM, and UM cell lines, respectively. Knockdown of ZEB1 increased (**C**) *FN1* but decreased (**D**) *PFN1* expression accordingly. (**E**) ChIP assays showed that ZEB1 bound to both *FN1* and *PFN1* promoters. Detected by scratch assays, (**F**) overexpression of ZEB1 in OCM1 increased cell migration while knockdown of ZEB1 in (**G**) OCM1 and in (**H**) C918 cells reduced their migration to cross the scratched gap. Yellow arrows indicate that semi-suspended cells detached from the monolayer culture are of amoeboid cell shape. (**I**) Kaplan-Meier survival curves of two large cohorts of total 63 and 53 primary UMs, respectively (microarray data sets GES22138 and GES44299, downloaded from NCBI database, Supplemental Tables 4 and 6), were generated and compared between ZEB1 high group (n = 21 or 18) and ZEB1 low group (n = 42 or 35). (**J**) Equal number of OCM1- and C918-vector controls and ZEB1sh knockdown cells (2 × 10^5^) were intravitreously injected into the nude mice and their 25-day grafted ocular tumors were examined for potential metastasis evaluation. (**K**) A representative image where potential metastatic cells are seen to be close to a blood vessel (BL) and surrounded by the normal liver cells (LC) 25 days after intravitreal injection of C918-vector control cells. Mean (M) ± standard deviation (SD). Student’s t-tests were performed. ‘**’ Indicates *p* ≤ 0.01. *** Indicates *p* ≤ 0.001. Yellow arrows indicate amoeboid-like cell morphology.

Adherent cells are anchored firmly by cell-cell and cell-extracellular matrix contacts through membrane adhesion molecules like E-cadherin (CDH1) for cell-cell conjunction, and integrins to connect the cytoskeleton to the matrix fibronectin (FN1), lacking such adherers would facilitate tumor cell dissemination both locally and in distance^47^. On the other hand, proteins involved in cytoskeletal structuring like profilin (PFN1) often play an important role in cellular locomotion^48^. We noticed that *CDH1* and *FN1* were decreased while *PFN1* was increased in UM and MetUM compared to NUM (Figs 3A and 8A,B), implying that UM cells might reduce cell-cell and cell-extracellular matrix connection allowing cells to separate from each other and to leave their original foci. It is of note that *FN1* was underexpressed in UM, but partially restored in MetUM (Fig. 8A), suggesting that MetUM might anchor more tightly to the extracellular matrix than UM. Knockdown of ZEB1 increased CDH1 and *FN1* while decreased *PFN1* expression in cultured UM cells (Figs 3E,L and 8C,D), suggesting that ZEB1 enhances loosening cell-cell contacts by transcriptionally repressing *CDH1* and *FN1* and promotes cell active locomotion by upregulating *PFN1* expression. ChIP assays showed that ZEB1 bound to and possibly repressed or transactivated *FN1* and *PFN1*, respectively (Fig. 8E). To measure UM cell mobility, we performed scratch assays on the confluent monolayer-cultured UM cells pretreated with mitomycin C to stop cell proliferation. We found that over-expression of ZEB1 in OCM1 significantly increased their mobility (Fig. 8F) while knockdown of ZEB1 in both OCM1 and C918 significantly reduced their cell mobility accordingly (Fig. 8G,H), confirming that ZEB1 enhances UM cell mobility. To test whether high expression of ZEB1 can be served as a biological marker for high metastasis thereby poor prognosis prediction in the clinic, we utilized two microarray data sets (GSE22138 and GSE44299) and the same classification based on their ZEB1 expression as described early (Supplemental Tables 4 and 6) and compared their Kaplan-Meier survival curves. The survival rates of both ZEB1^high^ groups were significantly (p < 0.001) lower than that of their counterpart ZEB1^low^ groups (Fig. 8I). Clearly, this data supports the notion that ZEB1^high^ in UM can serve as a single biological marker for poor prognosis. And indeed, within 25 days after cell IV injection, ZEB1^high^ C918-grafted tumors disseminated to the liver as compared to no dissemination in the ZEB1^low^ OCM1-grafted mice though knockdown of ZEB1 in C918 seemingly had no significant effect on metastasis (Fig. 8J,K, insert 8K1, Supplemental Table 5).

## Discussion

It is well documented in many malignant tumors of epithelium origin that EMT is critical for tumor cells to acquire more advanced capacities of local invasion, distant dissemination, and regeneration because EMT further transforms them into a more mobile, cell-cell independent, and stress resistant phenotype^47^. EMT is mainly resulted from an interaction of a group of E-box binding transcription factors including ZEB, SNAI, and TWIST families and greatly influenced by tumor microenvironment. For instance, both hypoxia and various cytokines like TGFβ can induce or enhance expression of ZEB1^49–55^. ZEB1 binds and thereby represses key epithelial cell-cell adhesion molecules like E-cadherin but promotes other key cytoskeletal elements like vimentin to induce EMT^56, 57^. Although EMT may not be as important for non-epithelium origin tumor, the EMT-TFs still play an important role in their progression. Here we provide evidence that ZEB1^high^ is clearly related to UM cell aggressiveness in agreement with the report on cutaneous melanomas^19^. It appears that the UM cell morphology does not correctly reflect the expression of the aforementioned EMT molecules and it is not known how UM cell morphology is controlled extrinsically and intrinsically. But, it is clear that EMT as an aggressiveness marker in carcinomas can be applied to judge aggressiveness of UMs molecularly. There is an obvious molecular switch from *SNAI* ^high^/*ID2* ^high^ to *ZEB1* ^high^/*TWIST1* ^high^ in accordance with the UM cell malignant advancement from being non-aggressive to becoming aggressive and ZEB1 appears to play a central role in coordinating other EMT-TFs to facilitate this switch. Since all the EMT-TFs and ID2 bind to the same E-box (CANNTG) sequence of the target genes it is not surprising that they might possess an antagonistic function by competition to bind the same sites. On the other hand, they may also bind thereby regulate each other, for example, ZEB1 binds and represses *SNAI1* and *ID2* (Fig. 3H,I,N). Using gain- and loss-of-function approaches we have clearly demonstrated that despite of its little effect on cell morphology, ZEB1 unquestionably promotes UM cell proliferation and invasiveness both *in vivo* and *in vitro* (Figs 5 and 7). In this regard, we favor the notion that EMT morphology switch itself is not as important per se, but EMT-TFs like ZEB1 are important in transcriptional modulation of target genes responsible for UM progression.

It is no doubt that ZEB1 promotes proliferation of both normal and transformed cells^6, 8^. In untransformed cells the expression of ZEB1 is restricted by tumor suppressor RB1^58^. But, ZEB1 also inactivates RB1 by binding to and repressing a group of CDKIs, particularly P16^INK4A^, critical in maintaining RB1 in an active form—dephosphorylated state^8^. Apparently, RB1 and ZEB1 form a regulatory loop in tumorigenesis. Mutation of RB1 deregulates ZEB1 restriction leading to superinduction of ZEB1 and constant cell division, whereas mutation of ZEB1 activates RB1 leading to cell senescence^6, 59^. In response to the critical shortening of the telomeres caused by constant cell division, another tumor suppressor TP53 would be activated to promote transcription of CDKIs particularly the P21^CIP1^ to activate RB1 to slow cycling down, and to prevent TP53 protein degradation and to transcript apoptosis-related genes like BAX leading to cell death^60^. Direct binding of ZEB1 to both *CDKN1A* and *CDKN2A* genes represses their expression and facilitates inactivation of RB1 and therefore exerts powerful tumorigenic effects on cell neoplastic transformation^6^. We also provided evidence here (Fig. 4) and elsewhere^37, 45^ that ZEB1 binds and represses *MITF*, a master TF for pigment cell differentiation to maintain the undifferentiated state of UM cells and to keep the switch towards more aggressive phenotype. In this regard, ZEB1 is a major oncogenic factor in UM progression by maintaining cell dedifferentiation and proliferation.

UM are highly malignant ocular tumors with high rates of metastasis^15^. However, cell metastasis is a complex process involving multiple cellular steps^47^. It is clear that the major cell-cell conjunction adherer CDH1 is an essential anchor for cells stay together in NUM, a significant reduction in CDH1 is a clear sign for UM and MetUM cells to be ready to detach from each other (Fig. 3A)^47^. Except for detaching from contacted cells, a tumor cell also needs de-anchor from the extracellular matrix in order to be able to leave. Fibronectin (FN1) is a major component secreted by the live cells to the extracellular matrix to anchor the cells locally to the cell cytoskeleton through the membrane protein integrins^61^. It appears that the expression levels of *FN1* are also correlated with cell anchoring ability as UM and UM cell lines express less *FN1* than NUM (Fig. 8A). Even if the tumor cells possess the abilities to detach, to de-anchor, and to break through the surrounding tissues they will not invade or/and metastasize without acquiring ability to travel. Migration of eukaryotic cells can be divided into two types: slow mesenchymal migration (crawling) and rapid amoeboid migration (sliding)^59^. In progression towards metastasis carcinoma cells first undergo a transition to mesenchymal migration and then amoeboid migration^26, 61, 62^. These two types of migration can be switch back and forth depending on the extrinsic conditions^26, 62^. We have noticed that both OCM1 and C918 cells may change their morphology to a small round amoeboid-like morphology at confluence particularly when the confluent culture is scratched (Fig. 8G,H, arrows) or when a suspended sphere touches down and derives new single cells that manifest a rapid migration behavior by crawling or flowing away from the original mass to distant sites as new colonies (Fig. 1G,H). Thus, it is not surprising that in the later stage of UM, the rapid growing epithelioid cells would eventually take over those less mitotic spindle cells in the UM of mixed phenotypes. This hypothesis is, at least partially, supported by our earlier observations that both epithelioid and spindle cells were produced in the spindle OCM1-derived grafts whereas only epithelioid cells was generated in the epithelioid C918-derived grafts (Fig. 1A‒F). We therefore speculate that epithelioid UM would be such a terminally defined phenotype with rapid proliferation, quick mobility, and high invasive and disseminating abilities. However, the mechanism underlying the UM morphology change and the question of whether this morphology change is related to the formation of cancer stem cells and thereby metastatic UMs, are not clear and subjected to further investigation.

## Methods

### Tumor samples

This study was approved by the Institutional Review Board of Central South University and adhered to the tenets of the Declaration of Helsinki. Primary tumors were collected at the time of enucleation. Written informed consent was obtained. Tumor samples were processed to make paraffin-embedded sections for H&E staining and immunostaining with desired antibodies.

### Animals

All athymic nude mice were purchased from the Jackson’s Laboratory (Cat# 002019), breeding, husbandry, and surgery procedures were in accordance with the Association for Research in Vision and Ophthalmology (ARVO) Statement for the Use of Animals in Ophthalmic and Vision Research and were approved by the Central South University or by University of Louisville Institutional Animal Care and Use Committee (IACUC) regulation.

### Cell culture

Dr. Klara Valyi-Nagy in the University of Illinois at Chicago generously provided two human uveal melanoma cell lines C918 and OCM1 whose authenticity was firmly validated^23^. UM cells were cultured in DMEM with 10% heat-inactivated fetal bovine serum (FBS) in a 37 °C and 5% CO_2_ incubator.

### Overexpression and Knockdown of ZEB1

The lentiviral constructs with a full-length cDNA of human *ZEB1* and 5 short hairpin interfering RNA (shRNA) sequences including 4 fragments against human *ZEB1* gene and 1 scramble fragment against nothing were purchased from Genechem Technologies (Shanghai, China), Shanghai GenePharma Co Ltd (Shanghai, China), respectively (Supplemental Table 1). OCM1 cells were transfected either with the ZEB1 overexpression plasmid (OCM1-ZEB1) or with the vector plasmid as a vector control and selected by a puromycin culture for 30 days. The lentivirus particles of the above 4 *ZEB1*-shRNAs and 1 scramble shRNA together with an EGFP gene were assembled in the laboratory as described previously^6^. Cells of both C918 and OCM1 were infected separately with the above 4 *ZEB1*-shRNA and the scrambled EGFP vector lentivirus as a vector control. The infected cells were purified by 2 μg/ml puromycin in culture for a week. Based on expression levels of EGFP and *ZEB1* detected by qPCR in C918 cells transduced with the above 4 *ZEB1*-shRNAs, we selected the *ZEB1* interfering nucleotide sequence 5′-AACAATACAAGAGGTTAAA-3′ for functional studies in both OCM1 and C918 cell lines. The overexpression or knockdown of ZEB1 was validated in the transduced cells by both qPCR and western blot (WB).

### Intravitreal (IV) and subcutaneous (SC) injection

We initially injected 2 × 10^5^ cells of both OCM1 and C918 cell lines intravitreously and subcutaneously into both eyes and rear flanks of 4 athymic nude mice of 3-month-old separately, total of 4 eyes and 4 flank foci of two mice for each cell line. This initial animal experiment was designed to evaluate how fast the grafted tumors would grow and whether there would be any metastasis in the liver. Therefore, all injected eyes and flanks were carefully examined every 2–3 days until day 13 after injection when some eye tumors were overgrown, thereby all 4 mice were euthanized. For ZEB1 overexpression analysis, 2 groups of 4 athymic nude mice of 6-week-old were utilized. One eye was IV injection of either OCM1-vector control or OCM1-ZEB1 cells whereas the other eye was IV injection of PBS as a sham control. For ZEB1 knockdown analysis, 4 groups of 2 athymic nude mice of 6-week old were utilized. Both eyes of the same nude mouse were injected the same cell line. Total 4 eyes of 2 nude mice were used for each cell line. For the cell implantation, all mice were first anesthetized by intraperitoneal (IP) injection of a mixture of 100 mg katamine and 10 mg xylazine per kg of the body weight, and then 2 μl of 2 × 10^5^ trypsinized single cells of indicated cell line in phosphate buffered saline (PBS) were delivered into the vitreous through the ora serrata or subcutaneous tissue of the rear flanks using a 30-gauge needle attached to a microsyringe. Antibiotic ointment was applied to the grafted eyes, and the grafted mice were allowed to recover on a heating pad before returned to their cages. PBS injections did not produce any tumor in the injected eyes whereas the grafted tumors were all visible in the implanted eyes by day 25 post injection of UM cell lines. Tumor growth was evaluated every 2‒3 days after cell implantation by observation with an ophthalmoscope and by measuring tumor size with a clipper. In 25 days after implantation when all the tumors were over grown, the mice were euthanatized by CO_2_, and the eyes were enucleated and fixed overnight in 10% neutral buffered formalin and then in 70% ethanol until further processed for embedding in paraffin. Sections at 10 μm were prepared for histopathological and immunostaining analyses. Pieces of the liver were also processed for possible metastasis evaluation.

### Cell invasiveness transwell assay

Cell invasion assays were performed using a 12-well transwell cell culture system with an upper chamber membrane of 8 μm pores. The transwell chamber membrane was coated with Matrigel at 1.5 mg/ml. 1 × 10^4^ cells in suspension were seeded on each upper chamber and cultured in a serum-free DMEM for 4 hours and then treated with 5 μg/ml mitomycin C for 2 hours at 37 °C and thereafter washed with PBS. The same medium but with 10% FBS was added to the lower chamber. After 24-hour culture at 37 °C and 5% CO_2_ the upper chamber cells passed through the dividing membrane to the lower chamber and adhered to the back of the membrane. The cells thereafter were fixed with 4% formaldehyde and stained with 0.5% crystal violet. All the cells on the upper side of the membrane were wiped away with a cotton swab with 70% ethanol solution. Three transwell invasion assays were performed for each cell lines. The number of invading cells on the backside of the membrane was pictured and counted with a Zeiss inverted microscope (Axiovert 200).

### Cell migration scratch assay

Monolayer cultured cells at confluence were treated with 5 μg/ml mitomycin C for 2 hours at 37 °C and then washed with PBS, followed by a straight scratch using a 200P pipette tip, and pictured under the inverted microscope and re-pictured every two hours at the same location.

### Immunofluorescence (IF)

Cells were cultured in 8-well chamber glass slides coated with 0.1% gelatin until confluence. They were fixed with 4% paraformaldehyde, rinsed with PBS, and blocked with 3% serum and 1% BSA for immunostaining using following primary antibodies: rabbit anti-ZEB1 (gift from Dr. Douglas Darling, 1:1000)^35^, mouse anti-Ki67 (BD Pharmingen, Cat# 550609, 1:50), mouse anti-E-cadherin (BD Biosciences, Cat# 610181), rabbit anti-N-cadherin (ThermoFisher, Cat# PA5-19486) together with an anti-rabbit IgG secondary antibody conjugated with Alexa fluor 568 (Invitrogen, Cat# A11011, 1:500), or an anti-mouse IgG second antibody conjugated with Alexa fluor 488 (Invitrogen, Cat# A11001, 1:500). Nuclei were counterstained with Hoechst dye (Invitrogen, Cat# H1399, 1:500), and images were captured by an invert fluorescent microscope.

### Immunohistochemistry (IHC)

Formalin-fixed and paraffin-embedded sections of human uveal melanomas were deparaffinized by xylene, and re-hydrated with series of ethanol solutions into final PBS. All sections were blocked with 3% serum isolated from the species where the primary antibody was raised plus 1% BSA. The same primary and secondary antibodies were used and images were captured as for above IF.

### Western blot (WB)

Cultured cells were collected in a cell lysis buffer (50 mM Tris/HCl, pH7.4, 100 mM NaCl, 0.5% Triton X-100, 0.5% NP-40), the supernatants were used as total extracted proteins after centrifuge at 13,000 rpm for 10 minutes for SDS-PAGE electrophoresis. The separated proteins were then transferred to a nitrocellulose membrane and followed by hybridization to the following antibodies: ZEB1 (1:2000)^35^, E-cadherin (BD Biosciences, Cat# 610181, 1:500). Blots were washed in PBS containing 0.1% Tween 20 and hybridized to goat anti-rabbit IgG-HRP (DAKO, 1:1000) or rabbit anti-mouse IgG-HRP (DAKO, 1:1000). Equal loading of protein samples was confirmed by probing the membranes with β-actin antibody (Sigma, Cat# A1978, 1:2000).

### RNA extraction and real-time quantitative PCR (qPCR)

Total RNA was extracted using TRIzol solution (Invitrogen). Complementary DNA was synthesized using the Invitrogen RT Kit according to the manufacturer’s protocol (Invitrogen). SYBR Green real-time quantitative PCR was performed using the Mx3000P Real-Time PCR System (Stratagene) as previously described^6^. PCR primer sequences were generated by a web-based program “Prime3” and synthesized by Integrated DNA Technologies (IDT) and shown in Supplemental Table 2. Three independent biological samples in technical triplicate were analyzed, and the PCR amplicons were validated by size on 1.5% agarose gel.

### Chromatin immunoprecipitation (ChIP)

ChIP assays were as described previously^6^ using formaldehyde to crosslink genomic DNA of C918 cells. The chromatin was mechanically sheared to an average length of 200–700 bps. Rabbit polyclonal antiserum for ZEB1^35^ was used for immunoprecipitation whereas equal amount of pre-immune serum was used as a background control (IgG). Immunoprecipitation with histone 3 antibody (H3) included in EpiTect ChIP antibody kit (Qiagen, Cat# GAH-2206) was used as a positive control. Sequences of primers for target promoters and the expected size of the PCR products are shown in Supplemental Table 3. ChIP-PCR programs were similar to that previously described for qPCR^6^, but with additional 1% BSA and 1% DMSO, and the PCR programs usually had a higher annealing temperature (e.g. 60–68 °C) and longer extension time (e.g. 1 minute).

### Microarray data analyses

For transcriptional expression profiling analyses, two microarray data sets were retrieved from National Center for Biotechnology Information (NCBI) database that include three samples of human normal melanocytes (NUM) (GSE62075)^31^, two samples of human uveal melanomas (UM—MM77 and MM81) and two samples of their metastatic tumors (MetUM—Mets MM77 and Mets MM81) (GSE59717)^32^. The raw intensity reads of all genes/spots were log2-transformed and normalized cross tumor samples and cross genes/spots. The selected genes were analyzed for transcriptional expression profiling after normalized to that of the house-keeping gene β-actin (*ACTB*). For correlation analyses of ZEB1 with the selected genes and tumor malignant properties, two additional microarray data sets (GSE22138 and GSE44299) of total 63 and 53 human primary UMs with patients’ information, respectively, were collected from NCBI database (Supplemental Tables 4 and 6)^24^. The raw intensity reads of all genes/spots were log2-transformed and normalized cross tumor samples and cross genes/spots. The tumor samples were sorted from high to low based on the expression levels of *ZEB1*, and divided into two groups: the top one third of tumor samples were assigned as *ZEB1*-high (n = 21 for GSE22138, n = 18 for GSE44299) whereas the bottom two third of tumor samples were assigned as *ZEB1*-low (n = 42 for GSE22138, n = 35 for GSE44299). An arbitrary number for each of the descriptive clinic traits in the patients’ information table (Supplemental Table 4) was assigned for quantitative analyses, such as ‘yes’ for ‘1’ and ‘no’ for ‘0’. *P* values to evaluate differences between groups for a specific trait were calculated by a two-tail, unpaired, and unequal variation Student’s t-test. Kaplan-Meier survival curves and *p* values for evaluating difference between groups were generated with the GraphPad Prism program.

### Statistical analyses

Comparisons between cell lines were assessed using a two-tail, unpaired, and unequal variation Student’s t-test. All values in the graphs are presented as means ± standard deviations. Three-star ‘***’ indicates p-value ≤ 0.001, two-star ‘**’ indicates p-value ≤ 0.01, whereas one-star ‘*’ indicates p-value ≤ 0.05. For *in vitro* studies including qPCR and cell growth rate calculations, results were obtained from at least 3 independent experiments of three technical replicates or otherwise indicated.

## Electronic supplementary material

Supplemental Tables
