# Supplementary material for: ZEB1 Regulates Multiple Oncogenic Components Involved in Uveal Melanoma Progression
Source: Sci Rep. 2017 Mar 3;7:45. doi: 10.1038/s41598-017-00079-x (PMC5428321; doi:10.1038/s41598-017-00079-x)
Supplement: Supplementary file 1 — Supplemental Tables [file 41598_2017_79_MOESM1_ESM.pdf]

ZEB1 Regulates Multiple Oncogenic Components Involved in Uveal Melanoma Progression

Yao Chen, Xiaoqin Lu, Diego E. Montoya-Durango, Yu-Hua Liu, Kevin C. Dean, Douglas S. Darling, Henry J. Kaplan, Douglas C. Dean, Ling Gao, Yongqing Liu

Supplemental Table 1. Human ZEB1 interference target sequence information

ID	Target sequence (5’-3’)	Start site	GC content (%)
ZEB1-RNAi (22902-1)	TGTTGTTCTGCCAACAGTT	1201	42.11
ZEB1-RNAi (22904-1)	GCGCAATAACGTTACAAAT	70	36.84
ZEB1-RNAi (22905-1)	AACAATACAAGAGGTTAAA	3706	26.32
ZEB1-RNAi (22906-1)	TGCCAATAAGCAAACGATT	2512	36.84
Scramble control	CAACAAGATGAAGAGCACCAA	N/A	40.90

Supplementary Table 2. Primers used for real-time quantitative PCR

Primer name	Sequence (5' - 3')	Tm ˚C	Amplicon (bp)
Hs ACTB LP	GGACTTCGAGCAAGAGATGG	55.3	234
Hs ACTB RP	AGCACTGTGTTGGCGTACAG	57.9	
Hs BAP1 LP	AAGCCTCATTTGCAAAGCCA	55.9	195
Hs BAP1 RP	GGTGCTGTCTCTCTCTCCTC	56.5	
Hs CDH1 LP	TTTCCACCAAAGTCACGCTG	55.9	162
Hs CDH1 RP	GGCAGCTGATGGGAGGAATA	56.8	
Hs CDH2 LP	ACAGTGGCCACCTACAAAGG	57.4	201
Hs CDH2 RP	CCGAGATGGGGTTGATAATG	52.9	
Hs CDKN1A LP	CAGCAGAGGAAGACCATGTG	55.6	153
Hs CDKN1A RP	GGCGTTTGGAGTGGTAGAAA	55.2	
Hs CDKN1B LP	CCGGCTAACTCTGAGGACAC	56.2	221
Hs CDKN1B RP	CTTCTGAGGCCAGGCTTCTT	55.1	
Hs CDKN2A LP	AGGGTTTTCGTGGTTCACAT	55.2	150
Hs CDKN2A RP	CTGCCCATCATCATGACCT	56.7	
Hs CDKN2B LP	GGTGAGAGTGGCAGGGTCT	56.3	174
Hs CDKN2B RP	CGGGGACTAGTGGAGAAGGT	56.7	
Hs CDKN2C LP	GGGCAGGTTCCCTTCATTAT	55.4	223
Hs CDKN2C RP	TGCACAAAATGGATTTGGAA	56.1	
Hs CDKN2D LP	ATGCTGCTGGAGGAGGTTC	57.6	158
Hs CDKN2D RP	CTGCCAAACATCATGACCTG	56.2	
Hs FN1 LP	GCAACGATCAGGACACAAGGAC	58.2	207
Hs FN1 RP	GGTGAGGCTGCGGTTGGTAA	60.2	
Hs ID2 LP	CGCATCCCACTATTGTCAGC	56.1	155
Hs ID2 RP	CAGTGCTTTGCTGTCATTTGA	54	
Hs MITF LP	AACTCATGCGTGAGCAGATG	55.5	202
Hs MITF RP	TACTTGGTGGGGTTTTCGAG	54.7	
Hs MMP1 LP	ATGCTGAAACCCTGAAGGTG	56.2	234
Hs MMP1 RP	CTGCTTGACCCTCAGAGACC	55.7	
Hs MMP11 LP	GCAACCGACAGAAGAGGTTC	55.9	135
Hs MMP11 RP	ATACCTTTAGGGCCTCTGCC	56.6	
Hs MMP3 LP	AAGTGGAGGAAAACCCACCT	54.1	215
Hs MMP3 RP	TTTCCAGGTCCATCAAAAGG	55	
Hs PFN1 LP	GTGGAACGCCTACATCGACAAC	58.2	154
Hs PFN1 RP	TTTGCCAACCAGGACACCCA	59.8	
Hs PLAU LP	TCACCACCAAAATGCTGTGT	55.2	223
Hs PLAU RP	AGGCCATTCTCTTCCTTGGT	56.2	
Hs SNAI1 LP	GAAAGGCCTTCAACTGCAAA	55.3	249
Hs SNAI1 RP	TGACATCTGAGTGGGTCTGG	56.1	
Hs SNAI2 LP	TCGGACCCACACATTACCTT	55.2	210
Hs SNAI2 RP	TTGGAGCAGTTTTTGCACTG	54.6	
Hs TWIST1 LP	CTCGGTCTGGAGGATGGAG	56.7	228
Hs TWIST1 RP	CCACGCCCTGTTTCTTTGAA	56	
Hs TWIST2 LP	TACAGCAAGAAGTCGAGCGA	56	207
Hs TWIST2 RP	CTTGCTCAGCTTGTCAGAGG	55.7	
Hs TYR LP	ATTGATTTTGCCCATGAAGC	51.9	251
Hs TYR RP	TCCTCCAATCGGCTACAGAC	56.3	
Hs TYRP1 LP	CCTTGCGCTTCTTCAATAGG	53.8	245

Hs TYRP1 RP	TGGCCCCAGTATTTCTTCTG	54.6	
Hs Vim LP	CGAGGAGAGCAGGATTTCTC	54.8	91
Hs Vim RP	GGTATCAACCAGAGGGAGTGA	55.8	
Hs ZEB1 LP	CTACAACAACAAGACACTGCTGT	55.4	176
Hs ZEB1 RP	TGTTCTTTCAGAGAGGTAAAGCG	54.8	
Hs ZEB2 LP	CAAGAGGCGCAAACAAGCC	57.7	128
Hs ZEB2 RP	GGTTGGCAATACCGTCATCC	56.2	

Supplementary Table 3. Primers used for ChIP assays

Primer name	Sequence (5' - 3')	Tm ˚C	Amplicon (bp)
Hs CDH1 PRMT LP	TAGAGGGTCACCGCGTCTAT	57.3	200
Hs CDH1 PRMT RP	TCACAGGTGCTTTGCAGTTC	55.9	
Hs CDH2 PRMT LP	TCCCCACCATTCTTCATCTC	54.1	147
Hs CDH2 PRMT RP	CGGAGGGAAGCCTAGAGTG	57	
Hs GAPDH PRMT LP	TACTAGCGGTTTTACGGGCG	57.2	166
Hs GAPDH PRMT RP	TCGAACAGGAGGAGCAGAGAGCGA	63.3	
Hs FN1 PRMT LP	AAAGAAAGGGAGCGGGATGGG	59.7	190
Hs FN1 PRMT RP	GGGTGGTGGTAGTGTTTGAGGA	58.9	
Hs ID2 PRMT LP	CGCCAGCCCCGCACTTACTG	63.7	268
Hs ID2 PRMT RP	GGCGGAAGGTGGCACGTCTA	62.2	
Hs MITF PRMT LP	CCAGGCATGAACACACATTC	54.3	185
Hs MITF PRMT RP	GGCAGACCTTGGTTTCCATA	54.8	
Hs MMP11 PRMT LP	ATGGAACAATCTCGGCTCAC	55	177
Hs MMP11 PRMT RP	AGGTCAGGAGTTGGAGAGCA	57.9	
Hs CDKN1A PRMT LP	TATTAGCTGGGCATGGTGGT	56.3	177
Hs CDKN1A PRMT RP	GCAGCCCTGGCTTTTTGTTT	57.2	
Hs PFN1 PRMT LP	TTCCTCCTGCTTTCTCCTTCCT	57.8	217
Hs PFN1 PRMT RP	CAGCCCAGACACCGAACTTTG	58.6	
Hs PLAU PRMT LP	ATATCTGGGGACTGCCACTG	56.5	170
Hs PLAU PRMT RP	CCAACTTGCCTAAGACTGCC	56.1	
Hs SNAI1 PRMT LP	CACCAAAGCACACTTCCCTT	55.8	186
Hs SNAI1 PRMT RP	CACCCGTTCCTTCCCTTATC	55.1	
Hs SNAI2 PRMT LP	ATTTGGTCTTTGTGCAAGGC	54.3	211
Hs SNAI2 PRMT RP	CTCTCTGGGAGCTAGGAGGG	58.3	
Hs TWIST1 PRMT LP	CACAATGCGGAGCCTAATTT	53.6	161
Hs TWIST1 PRMT RP	CACTCTTCGGTGGAAGGAAA	54.6	
Hs ZEB2 PRMT LP	CTCCCTACACACTCTCCCTT	55.5	197
Hs ZEB2 PRMT RP	AGCTGCATCTTAGGGCATGT	56.8	

Supplemental Table 4. Patients' information associated with the GSE22138 data set

UM #	age	gender	eye	tumor location	tumor diameter	tumor thickness (mm)	tumor cell type	retinal detachm ent	extrascleral extension	chromosome 3 status	months to endpoint	metastasis	ZEB1
**ZEB1 high group**													
MU151	47.43	female	right	posterior and on equator	17	13.5	NA	yes	NA	rtial monoson	52.57	yes	157.248
MU6	46.4	male	right	posterior to equator	15	14	mixed	yes	yes	disomy	64	yes	156.305
MU175	53.07	female	right	posterior to equator	16	6	NA	NA	yes	monosomy	68.37	yes	150.686
MU152	78.98	male	left	NA	NA	11.1	epithelioid	no	no	monosomy	39.69	no	98.5651
MU86	75.75	female	right	posterior and on equator	12	10	epithelioid	yes	NA	monosomy	8.67	yes	94.5485
MU131	55.68	female	left	on equator	18	11.7	epithelioid	yes	no	monosomy	27.3	yes	78.7187
MU185	48.46	male	left	on equator	12	10.5	NA	yes	no	monosomy	14.95	yes	77.0308
MU164	62.88	male	left	on equator	NA	11.5	epithelioid	no	no	monosomy	12.88	yes	71.5672
MU8	84.99	male	right	anterior to equator	20	7	NA	no	NA	monosomy	67.81	no	67.5097
MU40	54.81	female	left	on equator	13	12.7	mixed	yes	yes	monosomy	0.92	yes	62.9913
MU215	64.55	male	left	on equator	10	15	mixed	yes	no	monosomy	14.85	yes	56.6534
MU11	59.1	male	right	on equator	20.9	10.5	epithelioid	yes	no	monosomy	6.14	yes	56.4684
MU166	54.41	female	left	on equator	10	9.3	epithelioid	yes	no	monosomy	0.13	yes	53.8595
MU167	77.68	female	right	anterior to equator	11	11.8	epithelioid	no	no	monosomy	24.44	yes	48.9174
MU109	63.03	male	right	anterior to equator	10	11	epithelioid	no	no	monosomy	24.54	yes	45.4974
MU110	65.2	male	right	on equator	NA	12.8	mixed	yes	no	rtial monoson	35.78	yes	45.4974
MU3	67.57	male	left	on equator	10	11.3	epithelioid	NA	NA	NA	32.13	yes	43.3136
MU210	70.26	male	right	on equator	15	13	mixed	yes	no	disomy	6.18	yes	43.2109
MU66	65.62	male	right	on equator	16	13	NA	yes	no	monosomy	14.16	no	40.1843
MU207	50.17	male	right	on equator	10	9	epithelioid	yes	no	disomy	31.18	no	37.3749
MU212	50.17	male	left	on equator	19.6	13.6	epithelioid	yes	no	monosomy	17.61	yes	35.7135
**Average**	**61.72**			**0.48**	**14.19**	**11.35**	**0.84**	**0.74**	**0.18**	**0.80**	**26.87**	**0.81**	**72.47**
**ZEB1 low group**													
MU62	66.2	female	right	NA	9	8.9	mixed	no	NA	NA	120.64	no	33.9489
MU44	75.66	male	left	on equator	15	12.4	NA	yes	no	NA	6.54	yes	32.778
MU65	68.51	male	left	NA	10	8.9	NA	yes	no	monosomy	60.06	no	31.5272
MU211	80.66	male	left	on equator	18	11	NA	no	no	monosomy	9.3	no	31.0561
MU206	69.05	male	right	posterior to equator	NA	13	epithelioid	NA	no	monosomy	8.34	yes	26.4749
MU197	56.33	male	right	posterior to equator	NA	9.9	epithelioid	yes	n	monosomy	7.33	yes	25.8678
MU67	47.62	female	left	on equator	17	9.7	mixed	yes	no	disomy	67.98	no	25.826
MU107	62.62	male	left	on equator	13	13	mixed	no	no	monosomy	100.4	no	25.4691
MU1	76.54	female	right	NA	NA	12.2	mixed	no	NA	NA	73	no	23.0124
MU213	66.26	male	left	on equator	15	10.5	epithelioid	NA	no	disomy	31.74	no	22.6956
MU174	72.79	male	left	on equator	20	12.4	epithelioid	no	no	monosomy	67.75	no	19.4268
MU208	56.25	female	right	on equator	15	11	epithelioid	yes	no	disomy	67.38	no	17.7128
MU9	50.64	female	left	on equator	NA	8.3	mixed	yes	no	disomy	86.08	no	17.4965
MU38	60.8	male	left	posterior to equator	15	7.5	mixed	yes	NA	NA	16.76	yes	16.3265
MU12	52.33	male	left	on equator	19.4	13.7	mixed	yes	no	disomy	55.79	no	15.3068
MU173	56.17	female	left	on equator	19	12	NA	no	no	disomy	62.69	no	15.0328
MU195	62.12	male	left	posterior to equator	NA	17	mixed	yes	no	monosomy	18.89	yes	14.51
MU216	60.35	male	left	on equator	16	12.5	NA	yes	yes	disomy	36.9	yes	14.4937
MU47	45.97	male	right	on equator	9	10.4	mixed	yes	no	rtial monoson	89.82	no	14.4523
MU87	67.46	female	left	on equator	18	11.7	NA	yes	no	monosomy	49.02	yes	14.4196
MU209	82.79	female	right	on equator	17	13.2	NA	no	no	monosomy	31.54	no	14.2763
MU214	56.1	female	left	on equator	12	13.7	epithelioid	yes	no	monosomy	50.46	no	14.2359
MU130	57.69	male	left	posterior to equator	16	10.5	NA	yes	no	disomy	93.73	no	13.9956
MU10	38.67	male	right	on equator	11	10	NA	yes	no	disomy	85.09	no	13.701
MU4	61.82	male	right	on equator	20	10.9	NA	yes	NA	NA	0.39	yes	13.3754
MU111	40.31	male	right	on equator	18	11	NA	yes	no	disomy	55.75	no	13.1327
MU192	68.83	female	right	posterior and on equator	11	11.5	epithelioid	yes	no	NA	58.74	yes	12.1026
MU108	51.91	female	right	posterior to equator	15	11	mixed	no	no	rtial monoson	85.82	no	11.7344
MU89	72.29	male	left	NA	22	14	epithelioid	yes	no	rtial monoson	20.99	yes	10.9548
MU63	51.95	female	left	on equator	23	15	mixed	no	no	disomy	95.47	no	10.4232
MU14	70.09	male	right	on equator	13	13.8	mixed	yes	no	monosomy	7.92	yes	10.0871
MU129	28.56	female	left	on equator	17	13	mixed	no	NA	disomy	90.02	no	9.66282
MU13	32.94	male	right	on equator	15	10.1	mixed	no	no	disomy	55.23	no	9.43508
MU85	69.09	male	right	all over the eye	21	14	mixed	yes	NA	NA	7.06	yes	9.40896
MU64	59.41	male	left	posterior to equator	NA	12	NA	no	no	monosomy	25.13	no	8.70116
MU218	73.13	male	right	on equator	NA	13	NA	NA	no	monosomy	7.59	yes	8.58265
MU48	51.61	female	left	on equator	12	10.5	epithelioid	no	no	monosomy	21.78	yes	7.90258
MU153	77.95	female	right	on equator	16	11.7	epithelioid	no	no	monosomy	18.96	yes	7.60776
MU132	72.82	male	left	on equator	20	14	mixed	no	no	disomy	70.14	no	6.70883
MU217	65.72	female	left	on equator	20	14	mixed	no	no	monosomy	44.39	yes	6.35748
MU88	40.41	female	left	on equator	15	11.4	NA	yes	no	monosomy	28.88	yes	5.50691
MU205	68.36	male	right	on equator	17	11.5	mixed	no	yes	disomy	35.55	yes	4.26394
**Average**	**60.64**			**0.39**	**15.98**	**11.80**	**0.68**	**0.56**	**0.06**	**0.53**	**48.26**	**0.43**	**15.71**

Note: For quantitative analysis purpose, all the descriptive characteristics are assigned arbitrary numbers such as '1' for 'yes' while '0' for 'no'. For tumor location, any 'anterior'-related for '1', any 'posterior'-related for '0', and 'on equator' for '0.5'. For tumor cell type, '1' for 'epithelioid', '0.5' for 'mixed', '0' for 'spindle'. For chromosome 3 status, 'monosomy' for '1', 'partial monosomy' for '0.5', 'disomy' for '0'. 'NA' is not counted. For metastasis, '1' for 'yes' and '0' for 'no'.

Supplemental Table 5. Indices of UM cell-grafted tumor malignancy. Intravitreal injection of the indicated UM cell lines into both eyes of two athymic nude mice for 25 days when euthanized, the enucleated eyes and the liver tissues were fixed, paraffin-embedded, and sectioned at 10 µm for tumor property evaluation upon H&E staining. Arbitrary scale numbers from 0 to 3 for tumor size, for example, no tumor is ‘0’, small tumor is ‘1’ only seen by an ophthalmoscope, medium size tumor is ‘2’ already visible but still within the eye socket, big tumor is ‘3’ already out of the eye socket. The numbers 0, 0.5, and 1 for tissue invasion and metastasis are given to denote the severity of the grafted tumors.

**Graft #**	**Tissue**	**Tumor size**				**Invasion**			**Metastasis**		
		**Big**	**Medium**	**Small**	**No**	**Retina**	**Lens**	**Sclera**	**Yes**	**Possible**	**No**
**OCM1-vector #495**	**OS**		2			1	1	0			
	**OD**		2			1	1	0			
	**Liver**										0
**OCM1-vector #494**	**OS**		2			0.5	0.5	0			
	**OD**		2			0.5	0.5	0			
	**Liver**										0
**AVG**		**2**				**0.5**			**0**		
**STD**		**0**				**0.43**			**0**		
**OCM1-ZEB1sh #488**	**OS**				0	0	0	0			
	**OD**			1		0	0	0			
	**Liver**										0
**OCM1-ZEB1sh #489**	**OS**			1		0	0	0			
	**OD**			1		0	0	0			
	**Liver**										0
**AVG**		**0.75**				**0**			**0**		
**STD**		**0.5**				**0**			**0**		
**C918-vector #491**	**OS**	3				1	1	1			
	**OD**	3				1	1	1			
	**Liver**									0.5	
**C918-vector #490**	**OS**		2			1	1	1			
	**OD**		2			1	1	1			
	**Liver**									0.5	
**AVG**		**2.5**				**1**			**0.5**		
**STD**		**0.58**				**0**			**0**		
**C918-ZEB1sh #492**	**OS**		2			0.5	0.5	0			
	**OD**			1		0.5	0.5	0			
	**Liver**									0.5	
**C918-ZEB1sh #493**	**OS**		2			1	1	1			
	**OD**		2			1	1	1			
	**Liver**										0
**AVG**		**1.75**				**0.67**			**0.25**		
**STD**		**0.5**				**0.39**			**0.35**		

Note: OS=left eye; OD=right eye; AVG=Average; STD=standard deviation.

Supplemental Table 6. Patients' information associated with the GSE44299 data set

UM#	Gender	Survive (d)	Death	Metastasis	ZEB1
**ZEB1 high group**					
UM 26	m	2041	0	0	13.5542
UM 8	f	457	1	1	7.615825
UM 25	m	562	1	1	7.562569
UM 45	m	1151	1	1	7.271459
UM 61	m	1126	0	0	6.723591
UM 32	f	2284	0	0	6.071536
UM 22	m	792	1	1	5.395182
UM 7	m	1644	0	0	5.078291
UM 33	m	2952	0	0	4.198076
UM 37	m	943	1	1	3.890266
UM 54	f	822	1	1	3.861673
UM 58	f	1218	0	0	3.400497
UM 28	f	1186	1	1	3.399544
UM 63	m	668	1	1	2.66079
UM 24	f	1157	1	1	2.295758
UM 6	m	731	1	1	1.963174
UM 41	f	342	1	1	1.49318
UM 27	m	822	1	1	1.301496
**Average**		**1161.00**	**0.67**	**0.67**	**4.87**
**ZEB1 low group**					
UM 20	f	1614	0	0	1.016303
UM 30	f	1491	0	0	0.858666
UM 64	f	945	0	0	0.745469
UM 40	m	1003	1	1	0.514446
UM 59	m	1218	0	0	0.323337
UM 51	f	1310	0	0	0.297591
UM 12	m	2284	0	0	0.243418
UM 56	m	1218	0	0	0.222075
UM 19	f	1766	0	0	0.125164
UM 60	m	488	1	1	0.002033
UM 1	m	547	1	1	n.d.
UM 3	m	2799	0	0	n.d.
UM 4	m	548	1	1	n.d.
UM 9	m	60	1	1	n.d.
UM 10	f	457	0	0	n.d.
UM 13	m	1796	0	0	n.d.
UM 15	f	518	0	0	n.d.
UM 16	m	975	1	1	n.d.
UM 17	f	1278	1	1	n.d.
UM 18	m	2434	0	0	n.d.
UM 21	f	1583	0	0	n.d.
UM 29	f	1614	0	0	n.d.
UM 31	f	791	1	1	n.d.
UM 34	m	365	1	1	n.d.
UM 36	m	26	1	1	n.d.
UM 38	m	3105	0	0	n.d.
UM 39	f	2782	0	0	n.d.
UM 42	m	1413	0	0	n.d.
UM 44	m	1192	1	1	n.d.
UM 46	m	1338	0	0	n.d.
UM 48	f	995	1	1	n.d.
UM 50	m	699	0	0	n.d.
UM 52	f	820	0	0	n.d.
UM 53	m	1553	0	0	n.d.
UM 55	f	1249	0	0	n.d.
UM 57	f	1188	0	0	n.d.
UM 62	f	1035	0	0	n.d.
**Average**		**1256.68**	**0.32**	**0.32**	**0.43**
